# HSP90 Modulates T2R Bitter Taste Receptor Nitric Oxide Production and Innate Immune Responses in Human Airway Epithelial Cells and Macrophages

**DOI:** 10.3390/cells11091478

**Published:** 2022-04-27

**Authors:** Ryan M. Carey, Benjamin M. Hariri, Nithin D. Adappa, James N. Palmer, Robert J. Lee

**Affiliations:** 1 Department of Otorhinolaryngology—Head and Neck Surgery, Perelman School of Medicine, University of Pennsylvania, Philadelphia, PA 19104, USA; benjamin.hariri@gmail.com (B.M.H.); nithin.adappa@pennmedicine.upenn.edu (N.D.A.); james.palmer@pennmedicine.upenn.edu (J.N.P.); 2 Department of Physiology, Perelman School of Medicine, University of Pennsylvania, Philadelphia, PA 19104, USA

**Keywords:** bitter taste receptors, macrophages, airway epithelium, nitric oxide, calcium, cilia, heat shock proteins, innate immunity, sinusitis

## Abstract

Bitter taste receptors (T2Rs) are G protein-coupled receptors (GPCRs) expressed in various cell types including ciliated airway epithelial cells and macrophages. T2Rs in these two innate immune cell types are activated by bitter products, including those secreted by *Pseudomonas aeruginosa*, leading to Ca^2+^-dependent activation of endothelial nitric oxide (NO) synthase (eNOS). NO enhances mucociliary clearance and has direct antibacterial effects in ciliated epithelial cells. NO also increases phagocytosis by macrophages. Using biochemistry and live-cell imaging, we explored the role of heat shock protein 90 (HSP90) in regulating T2R-dependent NO pathways in primary sinonasal epithelial cells, primary monocyte-derived macrophages, and a human bronchiolar cell line (H441). Immunofluorescence showed that H441 cells express eNOS and T2Rs and that the bitter agonist denatonium benzoate activates NO production in a Ca^2+^- and HSP90-dependent manner in cells grown either as submerged cultures or at the air–liquid interface. In primary sinonasal epithelial cells, we determined that HSP90 inhibition reduces T2R-stimulated NO production and ciliary beating, which likely limits pathogen clearance. In primary monocyte-derived macrophages, we found that HSP-90 is integral to T2R-stimulated NO production and phagocytosis of FITC-labeled *Escherichia coli* and pHrodo-*Staphylococcus aureus*. Our study demonstrates that HSP90 serves as an innate immune modulator by regulating NO production downstream of T2R signaling by augmenting eNOS activation without impairing upstream Ca^2+^ signaling. These findings suggest that HSP90 plays an important role in airway antibacterial innate immunity and may be an important target in airway diseases such as chronic rhinosinusitis, asthma, or cystic fibrosis.

## 1. Introduction

Bitter taste receptors (also known as taste family 2 receptors, or T2Rs, encoded by *TAS2R* genes) are G protein-coupled receptors (GPCRs) used by the tongue to detect bitter compounds [1,2]. However, many of the 25 human T2R isoforms are also expressed in other organs [1,2,3,4], including the nose, sinuses, and lungs [5]. These receptors regulate diverse processes such as airway smooth muscle contraction [6,7,8,9] and innate immune responses in the oral epithelium [10]. T2R receptors are also expressed in immune cells such as monocytes and macrophages (MΦs) [11,12,13], which are important players in airway innate immunity [14,15]. In the airway epithelium, T2R isoforms 4, 14, 16, 38, and possibly others are expressed in bronchial and nasal motile cilia [5]. These T2Rs are activated in response to acyl-homoserine lactone (AHL) and quinolone quorum-sensing molecules secreted by the common airway pathogen *Pseudomonas aeruginosa* [5,16,17]. 

Activation of the T2Rs in sinonasal cilia or unprimed (M0) MΦs causes Ca^2+^-dependent activation of nitric oxide (NO) synthase (NOS) [5], likely the endothelial NOS (eNOS) isoform expressed in both airway ciliated cells [18,19,20,21,22] and M0 MΦs [23]. In ciliated cells, NO activates soluble guanylyl cyclase to produce cyclic GMP (cGMP). NO activates protein kinase G (PKG) to elevate ciliary frequency to increase mucociliary clearance, the major physical defense of the airway. The T2R-activated NO also directly diffuses into the airway surface liquid (ASL), where it can have antibacterial effects [5]. NO can damage bacterial cell walls and/or DNA [24,25]. NO can also inhibit the replication of many respiratory viruses, including influenza, parainfluenza, rhinovirus [26], and SARS-CoV-1 and -2 [27,28,29,30]. In MΦs, T2R-stimulated NO production and cGMP production acutely increase phagocytosis [31]. Thus, T2R to NO signaling may also be an important therapeutic target in infectious diseases beyond the airway. As NOS and NO have been implicated in chronic rhinosinusitis [32] as well as asthma and other lung diseases [33], a better understanding of the mechanisms of NOS activation in airway epithelial cells may have implications beyond T2Rs.

The importance of T2Rs in upper airway defense is supported by observations that patients homozygous for the AVI *TAS2R38* polymorphism, which renders the T2R38 receptor non-functional, have an increased frequency of Gram-negative bacterial infection [34], higher levels of sinonasal bacteria in general [35,36] and specifically biofilm-forming bacteria [37], a higher frequency of chronic rhinosinusitis [38,39,40,41], and worse outcomes after functional endoscopic sinus surgery [42]. One study has suggested that *TAS2R38* genetics may also play a role in cystic fibrosis (CF) *P. aeruginosa* infection [43], though other studies have suggested that *TAS2R38* may not be a modifier gene in CF [44,45]. Recently, the *TAS2R38* PAV (functional) genotype has been associated with a lower mortality of SARS-CoV-2 compared with the AVI (non-functional) genotype [46]. A better understanding of the role of T2R38 and other T2Rs in airway innate immunity is important for determining if and how to leverage these receptors as therapeutic targets or predictive biomarkers. An important component of this is to better understand the T2R signaling pathway.

In this study, we explored the role of heat shock protein 90 (HSP90) in T2R function in two types of cells important for airway innate immunity: ciliated epithelial cells and MΦs. The members of the HSP90 class of molecular chaperones are highly conserved and ubiquitously expressed [47]. In addition to promoting protein folding, HSP90 regulates signaling by facilitating trafficking or localization of signaling proteins and/or functioning as molecular scaffolds to bring signaling molecules together. Because HSP90 is critical for endothelial cell NO production via eNOS as well as alcohol-stimulated NO-driven cilia beating in the airway [48], we tested if HSP90 is involved in T2R-dependent NO generation. HSP90 proteins can facilitate eNOS activation via scaffolding of eNOS with activating kinases such as Akt or Ca^2+^-bound calmodulin (CaM) kinases [49,50,51,52,53]. HSP90 has been localized to the base of airway cell cilia [48,54,55], suggesting that HSP90 may be localized close to T2Rs in airway ciliated cells and may help facilitate their signal transduction to eNOS. HSP90 may even shuttle between the cilia base and the cilia axoneme in airway ciliated cells during alcohol exposure [48]. We hypothesized that HSP90 may likewise be involved in T2R/bacterial-driven NO production and regulate this innate immune pathway. Furthermore, it was recently proposed that HSP90 inhibition by geldanamycin can revert Th2- and Th17-induced airway epithelial goblet cell metaplasia [56]. If HSP90 inhibition reduces T2R NO responses, this may have unwanted effects of reducing T2R/NO-mediated innate immunity.

To test the requirement for HSP90 in T2R signaling, we combined biochemistry and live-cell imaging with a human bronchiolar cell line, primary sinonasal epithelial cells, and primary monocyte-derived MΦs. Results below reveal important molecular insights into the T2R signaling pathway in both airway epithelial and immune cells and identify a specific role for HSP90 in airway epithelial NO-mediated innate immunity. 

## 2. Materials and Methods

### 2.1. Cell Culture

HEK293T human embryonic kidney cells were obtained from ATCC (ATCC Cat# CRL-3216, RRID:CVCL_0063) and cultured in high-glucose DMEM (Thermo Fisher Scientific, Waltham, MA, USA) plus 10% FetalPlex serum substitute (Gemini Biosciences, West Sacramento, CA, USA) and 1× cell culture penicillin/streptomycin (Thermo Fisher Scientific) as described [57]. For transfection, cells were seeded onto 8-well chambered glass coverslips (CellVis, Mountain View, CA, USA; precoated with poly-D-lysine) and transfected with eNOS-RFP, Wt HSP90, and/or D88N HSP90, kindly provided by W. Sessa (plasmids # 22497 [RRID:Addgene_22497], #22487 [RRID:Addgene_22487], and/or #22480 [RRID:Addgene_22480], respectively; Addgene, Watertown, MA, USA). S1179D and S1179A eNOS-RFP was generated by site directed mutagenesis from Wt eNOS-RFP and verified by sequencing (University of Pennsylvania Penn Genomic Analysis Core DNA Sequencing Facility). Mutation of the S to D introduces a negative charge and acts as a phosphomimetic of phosphoserine, creating an eNOS that functions as if constitutively phosphorylated at S1179. Mutations of the S to A prevents phosphorylation at that site. Cells were stimulated with 10 µg/mL SC79 (Cayman Chemical, Ann Arbor, MI, USA) that was made from 10 mg/mL SC79 stock in DMSO. Control solutions had 0.1% DMSO as vehicle control. No effects of DMSO alone were observed. RFP fluorescence was used to verify similar transfection efficiencies in all experiments. 

H441 small airway human club-like epithelial cells (American Type Culture Collection [ATCC], Manassas, VA, USA; Cat# HTB-174, RRID:CVCL_1561) were cultured in Minimal Essential Medium (MEM; Thermo Fisher Scientific) with Earle’s salts plus 10% FetalPlex serum substitute and 1× cell culture penicillin/streptomycin. Cells were seeded and imaged on plastic 48-well plates as they stuck very poorly to glass. For air–liquid interface (ALI) cultures, H441 cells were seeded onto collagen-coated 1.1 cm^2^ Transwell filters (for intracellular DAF-FM) or 0.33 cm^2^ Transwell filters (for extracellular Daf-2) both with 0.4 µm pore size (transparent; Corning, Corning, NY, USA) and grown to confluence for 5 days before exposing the apical side to air, as described previously for 16HBE and Calu-3 cells [58,59]. H441 ALIs were switched to bronchial epithelial cell basal medium (BEBM; Lonza, Walkersville, MD, USA) plus Lonza Singlequot supplements (differentiation medium) upon confluence and exposure to air. When the culture medium was removed from the upper compartment, basolateral medium was changed to differentiation medium (1:1 DMEM:BEBM) containing hEGF (0.5 ng/ mL), epinephrine (5 ng/mL), BPE (0.13 mg/mL), hydrocortisone (0.5 ng/mL), insulin (5 ng/mL), triiodothyronine (6.5 ng/mL), and transferrin (0.5 ng/mL), supplemented with 100 U/mL penicillin, 100 g/mL streptomycin, 0.1 nM retinoic acid, and 2% NuSerum (BD Biosciences, San Jose, CA, USA) all from the Lonza Singlequot supplements as described [22]. Cells were fed from the basolateral side only with differentiation medium for ~21 days before use.

Submerged H441 cells were infected with Green GENIe-expressing BacMam (Montana Molecular, Bozeman, MT, USA) as per the manufacturer’s protocol for adherent cells; the medium was changed 4–6 h after infection to BacMam-free medium containing 2 mM NaButyrate to maintain expression. For siRNA, H441s were treated with Acell SMARTPool siRNAs (Horizon Discovery, Waterbeach, UK) for human eNOS (NOS3; Catalog ID:E-006490-00-0005), nNOS (NOS1; Catalog ID: E-009496-00-0005), PAR-2 (Catalog ID:E-005095-00-0005), or non-targeting pool (Catalog ID: D-001910-10-05) as per the manufacturer’s instructions and as previously described [31]. H441 cells were transfected with Wt or D88N HSP90 (Kindly provided by W. Sessa, Addgene plasmids #22487 or #22480, respectively) using lipofectamine 3000 and the specific H441 protocol provided on the Thermo Fisher Scientific website. We used 5 µL of lipofectamine and 2 µg DNA per 8-well chambered coverglass (CellVis), equating to 0.25 µg DNA/well, with each well approximately equivalent to a 48-well plate in surface area.

A549 human adenocarcinoma alveolar type-2-like cells (NCI-DTP Cat# A549, RRID:CVCL_0023) were identically transfected with GFP-eNOS (provided by W. Sessa, Addgene plasmid #22444; RRID:Addgene_22444) and/or mCherry-HSP90 (provided by D. Picard, Addgene plasmid #108223; RRID:Addgene_108223). Cells were cultured in Ham’s F12K medium (Thermo Fisher Scientific) containing 10% FetalPlex and 1% penicillin/streptomycin mix and imaged in 20 mM HEPES-buffered Hank’s Balanced Salt Solution (HBSS). Cells were transfected and imaged on 8-well chambered coverglass (uncoated) using 470/20 nM band pass (bp) filter (for GFP excitation), 490 lp dichroic beamsplitter, and 520/40 nm bp filter (for GFP emission) or 600/50 nm bp filter (for mCherry emission). Lack of bleed-through of GFP emission with the mCherry emission filter was observed in pilot experiments imaging GFP transfection only and is demonstrated with no fluorescence when GFP-eNOS is expressed with mCherry alone (described in the main text). Imaging of A549 cells was carried out using a 40× 0.75 NA objective on in Olympus (Tokyo, Japan) IX-83 microscope with Hammamatsu (Tokyo, Japan) Orca Flash 4.0 sCMOS camera and XCite 120 Boost LED illumination source (Excelitas, Waltham, MA, USA) and MetaMorph (Molecular Devices, San Jose, CA, USA).

Primary human M0 MΦs were cultured as previously described [31] in high-glucose RPMI2650 medium with 10% human serum and 1x cell culture penicillin/streptomycin. De-identified monocytes from healthy apheresis donors were obtained from the University of Pennsylvania Human Immunology core with written informed consent of every participant and institutional review board approval. Cells isolated from 10 different individuals were used. As all samples were de-identified for race, age, sex, etc., samples were used in a blinded fashion. MΦs were differentiated by adherence culture for 12 days in 8-well chamber slides (CellVis) as described [31]. Our prior studies suggest no differences in T2R responses among MΦs differentiated by adherence alone or by adherence plus M-CSF [31], and thus adherence only was used for these studies. MΦs were treated with Acell SMARTPool siRNAs as described [31]. 

Primary human nasal epithelial cells were obtained in accordance with The University of Pennsylvania guidelines regarding use of residual clinical material from patients undergoing sinonasal surgery at the University of Pennsylvania with institutional review board approval (#800614) and written informed consent from each patient in accordance with the U.S. Department of Health and Human Services code of federal regulation Title 45 CFR 46.116. Inclusion criteria were patients ≥ 18 years of age undergoing sinonasal surgery for sinonasal disease (CRS) or other procedures (e.g., trans-nasal approaches to the skull base). Exclusion criteria included history of systemic inheritable disease (e.g., granulomatosis with polyangiitis, cystic fibrosis, and systemic immunodeficiencies) or use of antibiotics, oral corticosteroids, or anti-biologics (e.g., Xolair) within one month of surgery. Individuals ≤ 18 years of age, pregnant women, and cognitively impaired persons were not included. Tissue was transported to the lab in saline on ice and mucosal tissue was immediately removed for cell isolation. 

Sinonasal epithelial cells were enzymatically dissociated and grown to confluence in proliferation medium (50% DMEM/Ham’s F-12 plus 50% BEBM plus Lonza Singlequot supplements) for 7 days [22]. Confluent cells were dissociated and seeded on Corning Transwells (0.33 cm^2^, 0.4 µm pore size; transparent; corning) coated with BSA, type I bovine collagen, and fibronectin (Corning). When culture medium was removed from the upper compartment, basolateral medium was changed to differentiation medium as described above for H441 ALIs. Primary ALI cultures were genotyped for *TAS2R38* PAV (functional) or AVI (non-functional) polymorphims [60,61] as described [22]. Cell identity was verified based airway epithelial morphology (formation of motile cilia, goblet cells, transepithelial electrical resistance, etc.) observed after differentiation.

### 2.2. Live-Cell Imaging of Ca^2+^, NO, and cGMP

Unless indicated, all regents were from MilliporeSigma (St. Louis, MO, USA). Adherent, submerged HEK293Ts in 20 mM HEPES-buffered Hank’s Balanced Salt solution (HBSS) were simultaneously loaded and stimulated for 30 min in the presence of 10 µM DAF-FM-diacetate (Thermo Fisher Scientific) ± SC79 (Cayman Chemical, Ann Arbor, MI, USA) as indicated. HEK293Ts were then immediately washed three times in HBSS and imaged as below. Submerged H441 cells were loaded for 30 min with 10 µM DAF-FM-diacetate (Thermo Fisher Scientific) in 20 mM HEPES-buffered HBSS supplemented with 1× MEM amino acids followed by washing with HBSS + 1× MEM amino acids. Calbryte 590 AM (AAT Bioquest, Sunnyvale, CA, USA) was loaded identically. When used, L-NAME (10 µM), D-NAME (10 µM), U73122 (1 µM), U73343 (1 µM), or geldanamycin (all from Cayman Chemical, Ann Arbor, MI, USA) were included in the loading solution as 30 min pretreatment; cPTIO (10 µM) was only added after loading. Cells were then washed out of DAF-FM into HBSS with the continued presence of inhibitor for the start of the experiment. Transwells were loaded with DAF-FM-diacetate for 60 min and placed into a glass-bottom 12-well dish (CellVis) prior to imaging. MΦs were loaded with 5 µM fura-2-AM or DAF-FM DA for 45 min as previously described [31]. Primary human ALIs were loaded for 90 min with DAF-FM-diacetate as previously described [22]. Denatonium benzoate, sodium benzoate, and phenylthiocarbamide were from Sigma Aldrich (St. Louis, MO, USA) and N-(acetyloxy)-3-nitrosothiovaline SNAP, BIIB021, and VER-155008 were from Cayman Chemical (St. Louis, MO, USA).

DAF-FM, fura-2, and cGMP were imaged as previously described [22,62]. DAF-FM was imaged on a TS100 microscope (20× 0.75 PlanApo objective for MΦs on glass and 10× 0.3 NA PlanFluor objective for H441 cells submerged on plastic or grown on Transwells; Nikon, Tokyo, Japan) GFP filter set, XCite 110 LED (Excelitas Technologies, Waltham, MA, USA), and Retiga R1 Camera (Teledyne QImaging, Surrey, BC, Canada). Calbryte 590 was imaged using the same microscope plus TRITC filter set and 10× 0.3 NA PlanFluor objective, as submerged H441s on plastic necessitated a longer working distance. Images were acquired using Micromanager [63]. Fura-2 was imaged using MetaFluor (Molecular Devices, Sunnyvale, CA, USA) and standard fura-2 dual excitation filter set on IX-83 microscope (20× 0.75 NA PlanApo objective for MΦs on glass, 10× 0.4 NA PlanApo objective for H441 cells on plastic; Olympus, Tokyo, Japan) equipped with a fluorescence xenon lamp (Sutter Lambda LS, Sutter Instruments, Novato, CA, USA), excitation and emission filter wheels (Sutter Lambda 2), and Orca Flash 4.0 sCMOS camera (Hamamatsu, Tokyo, Japan). Green GENIe cGMP construct was imaged using a FITC filter set, IX83 microscope, 10× 0.4 NA PlanApo objective, XCite 120Boost LED illumination, and MetaMorph. 

### 2.3. Measurement of Ciliary Beat Frequency (CBF) 

Whole-field CBF was measured using the Sisson-Ammons Video Analysis system [64] as previously described [22] at ~26–28 °C, with the exception of bacterial cHBSS experiments, which were carried out at room temperature. Cultures were imaged at 120 frames/s using a Leica DM-IL microscope (20×/0.8 NA objective) with Hoffman modulation contrast in a custom glass-bottom chamber. Experiments utilized Dulbecco’s PBS (+1.8 mM Ca^2+^) on the apical side and 20 mM HEPES-buffered Hank’s Balanced Salt Solution supplemented with 1× MEM vitamins and amino acids on the basolateral side. As typically performed with CBF measurements [22], changes in CBF were normalized to baseline CBF. This was validated by measurements of raw baseline CBF (in Hz) between control and experimental cultures showing no significant differences, as indicated in the text. 

### 2.4. Bacteria Culture

For ciliary beating experiments, PAO-1 (ATCC 15692) and PAO-JP2 (ΔlasI, ΔrhlI; Tc^r^, HgCl_2_^r^) [65,66] were cultured in LB medium as described [34]. Conditioned HBSS (cHBSS) was prepared by taking the pellet of an overnight culture and resuspending to OD 0.1 in HBSS and incubating overnight with shaking. We used cHBSS over conditioned LB due to the slight stimulatory effects of LB alone on CBF at dilutions > 10% [34]. After centrifuging (5000× *g*, 15 min, 4 °C) to pellet bacteria, cHBSS was filtered through a 0.2 µm filter then diluted as indicated with unconditioned (unmodified) HBSS.

For antibacterial assays (as described in [34]), *P. aeruginosa* strain PAO1 was grown to log phase (OD = 0.1) and resuspended in a buffer designed to mimic physiological nasal airway surface liquid (ASL) conditions (50% saline containing 1 mM HEPES and 0.5 mM glucose with pH = 6.5). Nasal ALIs were placed in 24-well plates in antibiotic-free F12K medium (Thermo Fisher Scientific) plus glutamate on the basolateral side. Bacteria in 30 µL of this solution were placed on the apical side of the ALI and allowed to settle for ~10 min, at which point the bulk ASL fluid was aspirated. After 2 h at 37 °C, residual bacteria were removed by washing. For live/dead staining, bacteria were incubated with 2× solution of BacLight Bacterial Viability Kit (Thermo Fisher Scientific) containing SYTO9 (to stain live cells) and propidium iodide (to stain dead cells). Control experiments were similarly performed with incubation of Transwell filters containing no nasal cells with bacteria in saline solution or saline plus anti-Gram-negative antibiotic colistin sulfate (10 µg/mL). Live/dead (green/red) ratio was subsequently quantified in a Spark 10M (Tecan, Männedorf, Switzerland) using 485 ± 10 nm excitation with 530 ± 12 nm and 620 ± 20 nm emission. CFUs counts were obtained by taking aliquots of the live dead mix, diluting with saline, and spotting on LB plates. 

### 2.5. Immunofluorescence (IF) Microscopy

IF was carried out as previously described [22]. ALI cultures were fixed in 4% formaldehyde for 20 min at room temperature, followed by blocking and permeabilization in phosphate-buffered saline (PBS) containing 1% bovine serum albumin (BSA), 5% normal donkey serum (NDS), 0.2% saponin, and 0.3% triton X-100 for 1 h at 4 °C. H441 cells were fixed in 4% formaldehyde for 20 min at room temp, followed by blocking and permeabilization in PBS containing 1% BSA, 5% NDS, 0.2% saponin, and 0.1% triton X-100 for 30 min at 4 °C. Primary antibody incubation (1:100 for anti-T2R antibodies, 1:250 for tubulin antibodies) were carried out at 4°C overnight. Incubation with AlexaFluor (AF)-labeled donkey anti-mouse and rabbit secondary antibody incubation (1:1000) was carried out for 2 h at 4 °C. Transwell filters were removed from the plastic mounting ring and mounted with Fluoroshield with DAPI (Abcam; Cambridge, MA, USA)). For co-staining of T2R14 and T2R38, Zenon antibody labeling kits (Thermo Fisher Scientific) were used to directly label primary antibodies with either AF546 or AF647 as described [22]. Images of ALIs were taken on an Olympus Fluoview confocal system with IX-73 microscope and 60× (1.4 NA) objective and analyzed in FIJI [67]. Images of submerged H441 cells were taken on an Olympus IX-83 microscope with 60× (1.4 NA) objective using Metamorph. Anti-T2R38 (ab130503; rabbit polyclonal; RRID:AB_11156286) and anti-beta-tubulin IV (ab11315; mouse monoclonal; RRID:AB_297919) antibodies were from Abcam. Anti-T2R14 (PA5-39710; rabbit polyclonal; RRID:AB_2556261) primary antibody and conjugated secondary antibodies (donkey anti-rabbit AlexaFluor 546 [RRID:AB_2534016] and donkey anti-mouse AlexaFluor 488 [RRID:AB_141607]) were from Thermo Fisher Scientific. Alpha-tubulin antibody was from Developmental Studies Hybridoma Bank (12G10; mouse monoclonal; University of Iowa, Iowa City; RRID:AB_1157911). Anti-eNOS antibody (NB-300-605; rabbit polyclonal; RRID:AB_10002794) was from Novus (Littleton, CO, USA). Immunofluorescence images were analyzed in FIJI [67] using only linear adjustments (min and max), set equally between images that are compared. Compared images were always taken with the same exposure, objective, and other camera and microscope settings. Both conventional (0 = black) and inverted (0 = white) lookup tables (LUTs) were shown in this study to illustrate localizations as clearly as possible, since inverted LUTs can be useful for visualizing high-dynamic-range fluorescence data. Inverted LUTs used were from ChrisLUTs FIJI package [68] (C. Leterrier, Neuropathophysiology Institute, Marseille University).

### 2.6. Phagocytosis Assays

Phagocytosis assays were carried out as descried [31]. MΦs were incubated with heat-killed FITC-labeled *Escherichia coli* at 250 µg/mL (strain K-12; reagents from Vybrant phagocytosis assay kit; Thermo Fisher Scientific; Cat # E2861) in phenol red-free, low-glucose DMEM (Thermo Fisher Scientific) ± denatonium benzoate or other agonists or inhibitors for 15 min at 37 °C. As we found that phagocytosis was negligible from temperatures of 4 °C up to room temp in these assays [31], we recorded fluorescence from living cells at room temperature immediately after the 15 min 37 °C incubation with FITC-*E. coli*. Extracellular FITC was quenched with trypan blue per the manufacturer’s instructions, and fluorescence was recorded on a Spark 10M plate reader (Tecan; 485 nm excitation, 535 nm emission). For representative micrograph shown, MΦs on glass were incubated as above, and extracellular FITC was quenched with trypan blue and cells were washed ≥5× in PBS to remove residual extracellular FITC-*E. coli*. Remaining adherent MΦs were fixed in 4% formaldehyde (Electron Microscopy Sciences, Hatfield, PA, USA) for 10 min followed by DAPI staining in mounting medium (Fluoroshield with DAPI, Abcam). FITC-E. coli were then imaged using standard FITC filter set (Semrock, Rochester, NY, USA) on an inverted Olympus IX-83 microscope with 20× (0.8 NA) objective, XCite 120LEDBoost illumination source, and Hammamatsu Orca Flash 4.0 sCMOS camera. 

Phagocytosis assays were also carried out similarly using 125 µg/mL pHrodo red-labeled *S. aureus* (strain Wood 46; Thermo Fisher Scientific, cat # A10010) [31]. As pHrodo dyes only fluoresce when particles are internalized into low pH endosomes (previously demonstrated in [31]), this assay does not require washing or quenching of the extracellular pHrodo *S. aureus*. MΦs were incubated with pHrodo-*S. aureus* for 30 min at 37 °C as described [31] with excitation at 555 nm and emission at 595 nm measured on the Tecan Spark 10M plate reader. Background measurements were made using wells containing fluorescent *S. aureus* in the absence of MΦs. Representative images were taken as above except using a standard TRITC filter set (Semrock). 

### 2.7. Data Analysis and Statistics 

Multiple comparisons were made with one-way ANOVA with Bonferroni (pre-selected pairwise comparisons), Tukey–Kramer (comparing all values), or Dunnett’s (comparing to control value) post-tests; *p* < 0.05 was considered statistically significant. Asterisks (* and **) indicate *p* < 0.05 and *p* < 0.01, respectively. All data in bar graphs are shown as the mean ± SEM with n derived from biological replicates (separate experiments conducted with different passage/patient cells on different days). Images shown for comparison were collected on the same day under identical conditions with identical min/max settings. No non-linear (e.g., gamma) adjustments were made to any images for either display or analysis. Raw unprocessed image data were analyzed in FIJI [67] and resulting numerical data were analyzed in Excel (Microsoft) and/or Prism (GraphPad software, La Jolla, CA, USA). All data used to generate bar graphs and traces are available upon request.

## 3. Results

### 3.1. HSP90 Inhibition Reduces Heterologously Expressed eNOS Function in HEK293Ts and A549s

To first determine if we could recapitulate prior results that HSP90 is important for eNOS function [51,53,69,70,71] in a reductionist model, we expressed eNOS-RFP in HEK293Ts, an eNOS null cell line [72]. We measured NO production using reactive nitrogen species (RNS)-sensitive dye DAF-FM over 30 min. One mechanism by which eNOS can be activated is phosphorylation at S1177 (S1179 in bovine eNOS). We found that expression of phosphomimetic S1179D mutated eNOS dramatically increased DAF-FM fluorescence compared with Wt eNOS-RFP or S1179A eNOS-RFP, which cannot be phosphorylated at that site (Supplementary Figure S1A,B). HSP90 inhibitor geldanamycin (10 µM; 30 min pretreatment then continued throughout the 30 min experiment) reduced DAF-FM fluorescence in S1179D eNOS-RFP-expressing cells. Supporting that the role of HSP90 inhibition, we also found that co-transfection of a dominant negative (DN) HSP90 isoform (D88N) [71] reduced DAF-FM fluorescence (Supplementary Figure S1B). These results support prior studies that HSP90 is important for the function of eNOS itself independent of upstream signaling. 

Small-molecule Akt activator SC79 induces eNOS phosphorylation and NO production in airway epithelial cells [73]. SC79 activated DAF-FM fluorescence increases in HEK293Ts transfected with Wt eNOS but not in untransfected cells (Supplementary Figure S1C). In Wt eNOS-transfected cells, SC79-induced DAF-FM fluorescence increases were reduced by Akt inhibition, co-transfection with dominant negative (K179M) Akt [74], HSP90 inhibitors geldanamycin or BIIB 021, or co-transfection of DN HSP90 (Supplementary Figure S1D,E). All together, these data suggest that HSP90 is important for eNOS-mediated NO production, supporting many prior studies [52,53,71]. When we transfected GFP-tagged eNOS [75] and mCherry-tagged HSP90 [76] into submerged A549 airway cells, Förster resonance energy transfer (FRET) data suggested that heterologously expressed HSP90 and eNOS closely co-localized in an airway cell line, and this association or close co-localization may increase during T2R stimulation (Supplementary Figure S2). 

### 3.2. HSP90 Inhibition Reduces Endogenous T2R-Stimulated eNOS Function in Submerged H441 Cells

We next wanted to test if HSP90 activity affects endogenous eNOS function when activated by endogenous T2R receptors. We started by examining if T2R stimulation activates NO production in H441 small airway epithelial cells, a club cell-like cell line that expresses eNOS similarly to primary bronchial cells [20,77]. H441 cells produce NO in response to estrogen and other types of stimulation [20,77,78,79,80]. We observed positive immunofluorescence (IF) for eNOS in submerged H441s compared with rabbit serum and fluorescent secondary alone (Supplementary Figure S3A–C), confirming that H441s express eNOS as demonstrated previously by others [20,77]. 

We also noted positive T2R4 and T2R46 immunofluorescence in submerged H441s (Supplementary Figure S3D–F). The rationale for examining T2R4 and 46 was that T2R4 localizes to nasal cilia [5,22] and T2R46 localizes to bronchial cilia [4]. Both are also expressed in human monocyte-derived MΦs [31]. Quantitative PCR (qPCR) of submerged H441s for the T2Rs responsive to denatonium benzoate supported expression of both T2R4 and T2R46, as well as T2R30 (formerly known as T2R47) and possibly T2R13 and T2R10 (Supplementary Figure S4A). 

All of these T2R isoforms (4, 46, 10, 13, and 30) are activated by the bitter compound denatonium benzoate, which activates eight out of the 25 human T2R isoforms [81,82]. The denatonium benzoate effective concentrations (ECs) for T2R4 and T2R46 are ~300 and ~30 µM, respectively, with an EC_50_ of ~240 µM reported for T2R46 in a heterologous HEK293T expression system. To test if denatonium benzoate activated NO production in submerged H441s, we loaded H441s with reactive nitrogen species-sensitive dye DAF-FM to track NO production, as performed previously in primary nasal cells [22]; 1 mM denatonium benzoate increased in intracellular DAF-FM fluorescence that was inhibited by NO scavenger carboxy-PTIO, NOS inhibitor L-NAME, eNOS siRNA, phospholipase C (PLC) inhibitor U73122 (Supplementary Figure S4B–E). DAF-FM increases in response to T2R agonists denatonium, quinine and thujone (1 mM), which activate T2Rs in bronchial [4] and nasal cilia [83], were reduced in cells transfected with a dominant negative HSP90 beta (D88N-HSP90; Supplementary Figure S4F) [71]. Denatonium-induced DAF-FM fluorescence increases were also reduced ≥50% by pretreatment (1 h) with 10 µM HSP-90 inhibitor geldanamycin (Supplementary Figure S4G), suggesting that denatonium-induced (likely T2R-induced) NO production requires HSP90 activity. There was no alteration of the denatonium-induced Ca^2+^ responses with this concentration of geldanamycin (Supplementary Figure S4H), suggesting that the role of HSP90 is likely downstream of Ca^2+^ signaling. 

NO increases intracellular cGMP by activating soluble guanylyl cyclase. We tested if denatonium stimulation increased cGMP using a cGMP fluorescent biosensor (Green GENie; Montana Molecular). Denatonium caused an increase in cGMP (decrease in Green GENIe F/F_o_; plotted inversely in Supplementary Figure S5A,B) that was likewise inhibited by blockade of NOS activity by L-NAME (Supplementary Figure S5A,C) or geldanamycin pretreatment (Supplementary Figure S5B,C). These results all suggest that full activation of eNOS and downstream cGMP production during T2R agonist stimulation in H441 cells requires HSP90 function. 

### 3.3. HSP90 Inhibition Reduces Endogenous T2R-Stimulated eNOS Function in H441 Monolayers Cultured at the Air–Liquid Interface

**Figure 1:**
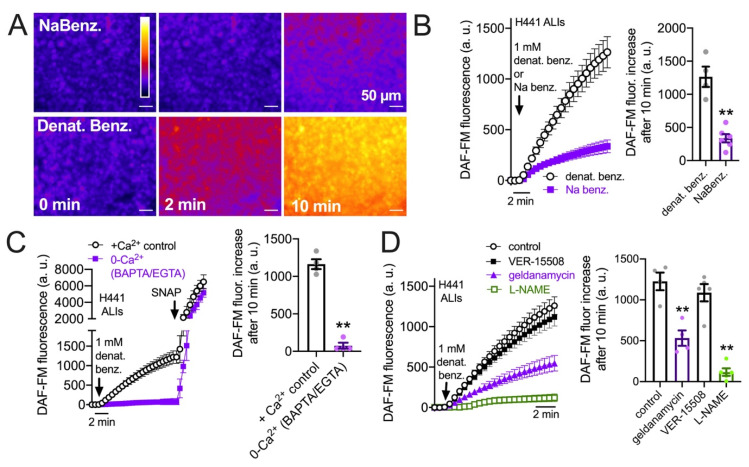
HSP90 inhibition reduces T2R-stimulated intracellular NO production in H441 cells grown at the air–liquid interface (ALI). (**A**)*:* Representative image of DAF-FM-loaded H441 ALIs stimulated for 10 min with 1 mM sodium benzoate (NaBenz.) or denatonium benzoate (denat benz.); fluorescence increased with denatonium benzoate but not sodium benzoate. (**B**)*:* Average trace and bar graph (mean ± SEM) of four experiments as in (**A**). Significance determined by Student’s *t*-test; ** *p* < 0.01. (**C**)*:* Average trace and bar graph (mean ± SEM of three experiments) showing response in cultures pre-loaded with BAPTA-AM and stimulated in the absence of extracellular Ca^2+^ (0-Ca^2+^_o_) vs. control cultures pre-incubated with 0.1% DMSO only and stimulated in the presence of extracellular Ca^2+^. (**D**)*:* Denatonium-induced DAF-FM fluorescence increases in H441 ALIs were inhibited by pretreatment with geldanamycin or L-NAME but not HSP70 inhibitor VER-15508. Average trace and bar graph of results from four independent experiments are shown. Significance determined by one-way ANOVA with Dunnett’s post-test comparing all values to control (denatonium only); ** *p* < 0.01.

While T2R/Ca^2+^-activated NO generation data support a role for HSP90 in submerged cells, submerged cells do not accurately reflect the polarized airway epithelium. We thus also grew H441 cell monolayers at the air–liquid interface (ALI), a more physiological cell culture model for airway epithelial cells than submersion. H441 ALIs have been used to study ion transport and barrier function [84,85,86,87,88]. Denatonium benzoate, but not sodium benzoate, activated NO production in H441 ALIs, measured by intracellular DAF-FM fluorescence (Figure 1A,B). While sodium benzoate does activate some T2Rs, it activates a subset (T2R14 and T2R16) distinct from denatonium benzoate with much lower affinity [81]. Thus, we use sodium benzoate as a control here for osmotic effects and potential pH effects due to permeation of the benzoate moiety. Denatonium-induced DAF-FM fluorescence increases were completely blocked by Ca^2+^ chelation by intracellular BAPTA-loading (45 min, 10 µM) plus 0-Ca^2+^ extracellular buffer (containing 2 mM EGTA to chelate trace calcium; Figure 1C). This suggests a requirement for Ca^2+^ signaling. Like submerged cells, the DAF-FM response was also reduced by 45 min pretreatment with HSP90 inhibitor geldanamycin (10 µM) or NOS inhibitor L-NAME (10 µM) but not HSP70 inhibitor VER-155008 (10 µM; Figure 1D). Thus, HSP90 inhibition reduces T2R-mediated NO production in H441 ALIs. 

**Figure 2:**
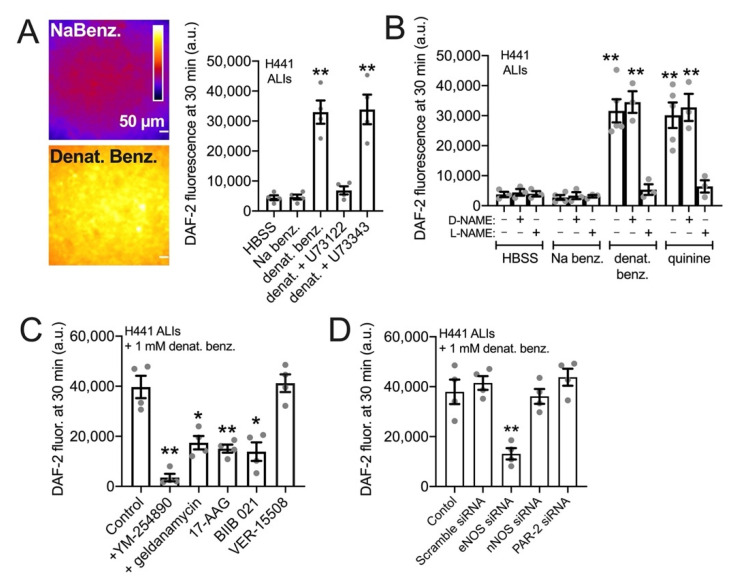
HSP90 inhibition reduces T2R-stimulated NO diffusion into the airway surface liquid (ASL) in H441 cells grown at the air–liquid interface (ALI) (**A**): Representative images and bar graph of 4 independent experiments of fluorescence at the apical plane of ALI when 100 µL of solution containing cell impermeable DAF-2 was placed on top (1.1 cm^2^ Transwell) either containing sodium benzoate (top) or denatonium benzoate (bottom). Cultures were either pretreated with 0.1% DMSO (vehicle control), 10 µM PLC inhibitor U73122, or 10 µM inactive analogue U73343 prior to the experiment. Significance determined by one-way ANOVA with Dunnett’s post-test comparing all values to HBSS only control. (**B**): Bar graph of experiments performed as in (**A**) but testing inhibition of denatonium-induced or quinine-induced ASL DAF-2 fluorescence ± NOS inhibitor L-NAME or inactive D-NAME (10 µM). Bar graph shows the mean ± SEM of 3–5 independent experiments imaged at identical conditions. Significance by one-way ANOVA with Bonferroni post-test comparing all values to respective HBSS control; ** *p* < 0.01. (**C**)*:* Denatonium-stimulated H441 DAF-2 ASL fluorescence increases were reduced in the presence of GPCR signaling inhibitor YM254890 or HSP90 inhibitors geldanamycin, 17-AAG, or BIIB 021. HSP70 inhibitor VER-15508 had no effect. Bar graph shows the mean ± SEM of four independent experiments. Significance by one-way ANOVA with Dunnett’s post-test comparing all values to control (0.1% DMSO only); * *p* < 0.05 and ** *p* < 0.01. (**D**): H441s were treated with siRNA as described in the methods. ASL DAF-2 responses during denatonium stimulation were reduced by eNOS siRNA but not with scramble, nNOS, or PAR-2 siRNA. Bar graph shows the mean ± SEM of four independent experiments (separate siRNA transfections). Significance by one-way ANOVA with Dunnett’s post-test comparing all values to control; ** *p* < 0.01.

NO is a highly diffusive gas that can rapidly diffuse across cell membranes [89]. When a small volume (100 uL) of cell impermeant NO indicator DAF-2 (10 µM) was placed on top of the H441 ALIs in the presence of denatonium benzoate or sodium benzoate for 30 min, we observed a 3-fold higher fluorescence of apical DAF-2 in denatonium-treated cultures (Figure 2A). This suggests that NO produced can diffuse into the airway surface liquid, as previously show in primary sinonasal ALIs [34]. The denatonium-induced DAF-2 fluorescence increase was reduced by PLC inhibitor U73122 but not inactive U73343 (Figure 2A), suggesting that it depended on GPCR signaling. We found that T2R agonists denatonium benzoate and quinine, but not sodium benzoate, both increased apical DAF-2 fluorescence in a NOS-dependent manner, as responses were inhibited by L-NAME but not D-NAME (Figure 2B). These DAF-2 responses, likely reflecting NO diffusion into the ASL, were blocked by pretreatment (10 µM, 45 min) with GPCR G protein inhibitor YM-254890 [90,91,92] or HSP90 inhibitors geldanamycin, 17-AAG, or BIIB 021 (Figure 2C) but were not blocked by HSP70 inhibitor VER-15508. Like submerged H441s, we saw inhibition with eNOS siRNA but not with scramble, nNOS, or PAR-2 siRNA (Figure 2D).

### 3.4. HSP90 Inhibition Reduces T2R-Driven NO Production in Primary Nasal Epithelial Air–Liquid Interface Cultures

**Figure 3:**
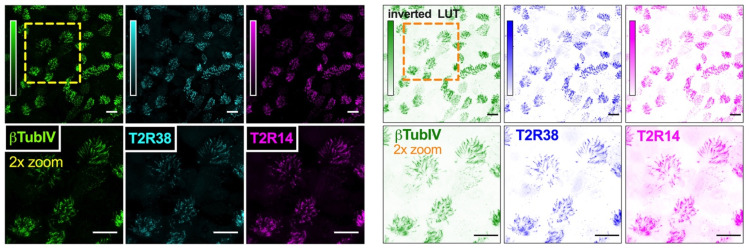
T2R bitter taste receptor expression in airway cell cilia. Representative images of cilia marker β-tubulin IV (green), T2R38 (cyan) and T2R14 (magenta) immunofluorescence in an apical confocal section of primary human sinonasal ALI. (**Left**) shows conventional look-up table (LUT) and (**right**) shows inverted LUT. Scale bar is 20 µm.

**Figure 4:**
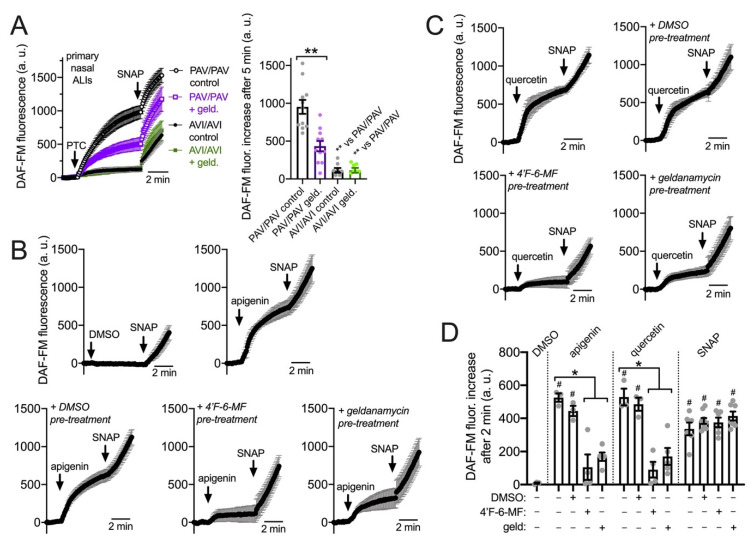
HSP90 inhibition reduces T2R-stimulated intracellular NO production in primary sinonasal epithelial cells grown at the air–liquid interface (ALI). (**A**): Intracellular DAF-FM increases were measured in response to T2R38-agonist PTC (1 mM) followed by NO donor SNAP (25 µM) as positive control. PTC stimulated NO production in ALIs from PAV/PAV (homozygous functional T2R38) but not AVI/AVI (homozygous non-functional T2R38) ALIs (nonfunctional T2R38) patients. Geldanamycin pretreatment inhibited the NO production in PAV/PAV ALIs. Trace and bar graph show the mean ± SEM of 8–10 experiments per condition using ALIs from 4–5 patients. Significance determined by one-way ANOVA with Tukey–Kramer post-test comparing all values; ** *p* < 0.01. (**B**): Traces of DAF-FM fluorescence in PAV/AVI (heterozygous T2R38) cultures stimulated with T2R14/39 agonist apigenin (100 µM) shown with 0.1% DMSO vehicle control. Pretreatment with T2R14/39 antagonist 4′-fluoro-6-methoxyflavanone (4′-F-6-MF) or HSP90 inhibitor geldanamycin but not 0.1% DMSO (inhibitor vehicle control) reduced apigenin-induced but not SNAP-induced DAF-FM fluorescence increases. (**C**): Traces of DAF-FM fluorescence in PAV/AVI (heterozygous T2R38) cultures stimulated with T2R14 agonist quercetin (50 µM). Pretreatment with T2R14/39 antagonist 4′-fluoro-6-methoxyflavanone (4′-F-6-MF) or HSP90 inhibitor geldanamycin but not 0.1% DMSO (inhibitor vehicle control) reduced quercetin-induced but not SNAP-induced DAF-FM fluorescence increases. (**D**): Bar graph of intracellular DAF-FM fluorescence increases after 2 min stimulation from experiments as in (**C**,**D**). Stimulation (DMSO vehicle control, apigenin, quercetin, or SNAP) listed on top and pretreatment (DMSO vehicle control, 4′-F-6-MF, or geldanamycin) listed on the bottom. Each data point is an independent experiment (*n* = 4–8 per condition). Significance by Bonferroni post-test; * *p* < 0.05 vs. bracketed bars; ^#^ *p* < 0.05 vs. DMSO alone.

We examined NO production using DAF-FM in primary sinonasal cells grown from residual surgical material and differentiated at ALI as described [22]. These cells express T2R receptors in apical motile cilia (Figure 3 and [22]). Note that, unlike H441 assays, denatonium benzoate was not used in primary nasal cell assays. While primary bronchial ciliated cells respond to denatonium benzoate [4], nasal ciliated cells do not [34], likely due to differential T2R isoform expression between bronchial and nasal cells. For primary nasal cells, we instead used the T2R38-specific agonist phenylthiocarbamide (PTC) [60,61]. We took advantage of primary ALIs genotyped for functional (PAV) or non-functional (AVI) polymorphisms in *TAS2R38* encoding the T2R38 receptor [34,61,93]; the AVI/AVI *TAS2R38* cultures are de facto human T2R38 knock outs. Homozygous PAV/PAV *TAS2R38* cells produced NO in response to 1 mM PTC while AVI/AVI cells did not (Figure 4A). The NO produced during PTC stimulation was inhibited by geldanamycin (10 µM, 45 min pretreatment; Figure 4A).

Apigenin, a T2R14 and 39 agonist [22,94], stimulates NO production and ciliary beat frequency increases in primary nasal ALIs via T2R14 [22]. Apigenin (100 µM) stimulation increased DAF-FM fluorescence in primary nasal ALIs that was reduced by pretreatment with T2R14/39 antagonist 4′-fluoro-6-methoxyflavanone (50 µM; 45 min [22]) as well as HSP90 inhibitor geldanamycin (10 µM 45 min; Figure 4B). We also tested quercetin, another plant flavonoid shown to be a T2R14 agonist in heterologous expression assays [95]. While quercetin was previously shown to increase (CBF) [96] and reduce cAMP signaling [97], a mechanism for these effects was not elucidated. As T2Rs also decrease cAMP through inhibitory G protein signaling in airway cells [98], we hypothesized that quercetin may act as a T2R agonist in airway epithelial cells. Quercetin (50 µM) stimulation likewise increased DAF-FM fluorescence that was blocked by 4′-fluoro-6-methoxyflavanone or geldanamycin (Figure 4C). Apigenin and quercetin-stimulated DAF-FM fluorescence responses are summarized in Figure 4D. 

**Figure 5:**
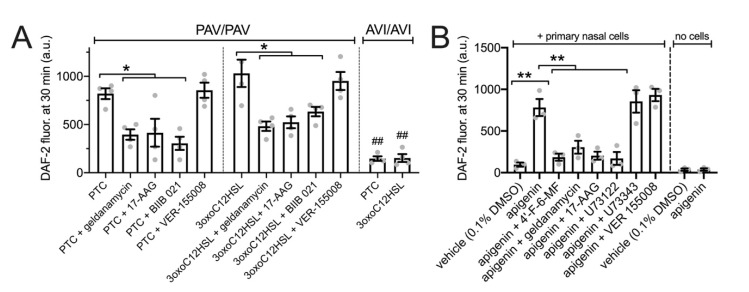
HSP90 inhibition reduces T2R-stimulated NO diffusion into the airway surface liquid (ASL) in primary sinonasal epithelial cells grown at the air–liquid interface (ALI) Experiments were performed as in Figure 2 to measure NO diffusion into the ASL but with primary nasal ALIs. (**A**): PTC (500 µM) or 3oxoC12HSL (100 µM) stimulated extracellular DAF-2 fluorescence in PAV/PAV and AVI/AVI cultures, as indicated. PAV/PAV cultures were also pretreated with HSP90 inhibitors geldanamycin, 17-AAG, or BIIB 021 or HSP70 inhibitor VER-155008. (**B**): shows experiments with apigenin ± 4′-F-6-MF, geldanamycin, 17-AAG, or PLC inhibitor U73122 and inactive analogue U73343. Control Transwells containing no cells were similarly incubated with vehicle only or apigenin to test for any cell-independent reaction of apigenin with DAF-2. Significance by one way ANOVA with Bonferroni post-test; * *p* < 0.05 vs. bracketed bars; ** *p* < 0.01 vs. bracketed bars; ^##^ *p* < 0.05 for the same condition in PAV/PAV vs AVI/AVI cultures.

We also performed similar assays as performed with H441s in Figure 5 to measure NO diffusion into the ASL using 30 µL of DAF-2 solution overlaid onto the primary nasal ALIs. Both PTC (1 mM) and *P. aeruginosa* quorum-sensing molecule 3oxoC12HSL (100 µM) increased apical surface DAF-2 fluorescence in a manner that was T2R38 dependent as it occurred in PAV/PAV (functional T2R38 homozygous) cultures but not AVI/AVI (non-functional T2R38 homozygous) cultures (Figure 5A). PTC- and 3oxoC12HSL-induced increases in DAF-2 fluorescence were inhibited by geldanamycin, 17-AAG, or BIIB 021 but not VER-15008 (all 10 µM for 45 min pretreatment; Figure 5A). Apigenin (100 µM) increased apical DAF-2 fluorescence in a manner that was inhibited by T2R14 antagonist 4′-fluoro-6-methoxyflavanone [99] (50 µM for 45 min pretreatment; Figure 5B) or PLC inhibitor U73122 (10 µM for 45 min pretreatment; Figure 5B). Apigenin-stimulated DAF-2 increases were also reduced by HSP90 inhibitors geldanamycin or 17-AAG but not by HSP70 inhibitor VER 155008 (all 10 µM for 45 min pretreatment) (Figure 5B). Phospholipase C (PLC) inhibitor U73122 (10 µM for 45 min pretreatment) also inhibited the apigenin response while inactive analogue U74434 had no effect. As a control, when apigenin or vehicle was incubated in DAF-2 solution in the absence of cells (just plastic Transwells), no differences in DAF-2 fluorescence were observed (Figure 5B).

### 3.5. HSP90 Inhibition Reduces T2R/NO-Driven Nasal Ciliary Beating

**Figure 6:**
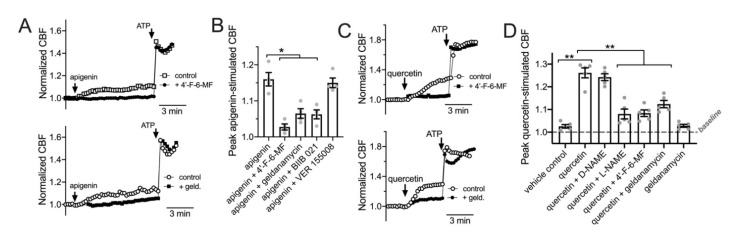
HSP90 inhibition reduces T2R-stimulated ciliary beating in primary sinonasal epithelial cells. (**A**): Left shows representative normalized CBF responses (representative experiments shown) to T2R14/39 agonist apigenin in human primary sinonasal ALIs ± T2R14/39 inhibitor 4′-fluoro-6-methoxyflavanone. Right shows normalized CBF responses (representative experiments shown) to apigenin ± geldanamycin (10 µM; 5 min pretreatment). Mean baseline CBF was not with vehicle or 4′-fluoro-6-methoxyflavanone pretreatment (7.5 ± 1.1 Hz or 8.2 ± 0.9 Hz, respectively; not significant by Students’ *t*-test). Mean baseline CBF was also not different before or after vehicle or geldanamycin pretreatment (6.9 ± 1.7 Hz or 7.9 ± 1.2 Hz, respectively; not significant by Students’ *t*-test). (**B**): Bar graph of the mean ± SEM of CBF responses from five independent experiments as shown in (**A**) using ALIs from four different patients. Significance determined by one-way ANOVA with Bonferroni post-test; * *p* < 0.05. (**C**): Left shows representative normalized CBF responses (representative experiments shown) to T2R14/39 agonist quercetin in human ALIs ± T2R14/39 inhibitor 4′-fluoro-6-methoxyflavanone. Mean baseline CBF was not with vehicle or 4′-fluoro-6-methoxyflavanone pretreatment (7.3 ± 1.2 Hz or 7.9 ± 0.6 Hz, respectively; not significant by Students’ *t*-test). Right shows normalized CBF responses (representative experiments shown) to quercetin ± geldanamycin (10 µM; 5 min pretreatment). Mean baseline CBF was not different before or after vehicle or geldanamycin pretreatment (7.4 ± 1.3 Hz or 7.0 ± 0.9 Hz, respectively; not significant by Students’ *t*-test). (**D**): Bar graph of the mean ± SEM of CBF responses from five independent experiments as shown in *C* using ALIs from five different patients. Significance determined by one-way ANOVA with Bonferroni post-test; ** *p* < 0.01.

Data in Figure 4 and Figure 5 suggest that HSP90 function is required for NO production during T2R38 or T2R14 activation in primary nasal epithelial cells. We tested if this affected ciliary beat frequency (CBF) using the T2R14 agonist apigenin. As previously described [5,22], apigenin increased ciliary beat frequency ~10–15% over 10 min. This was blocked by T2R14 antagonist 4′-fluoro-6-methoxyfavanone or HSP90 inhibitors geldanamycin or BIIB 021 (10 min pretreatment, 10 µM) but not by HSP70 inhibitor VER 1555008 (Figure 6A,B). Thus, HSP90 inhibitors reduced apigenin-stimulated T2R14 CBF responses. We also observed a ~30% increase in CBF with apical application of 25 µM quercetin (Figure 6C) that was reduced by the T2R14 inhibitor 4′-fluoro-6-methoxyflavanone [99] or geldanamycin. There was no inhibition of CBF increases in response to purinergic agonist ATP (50 µM; Figure 6A,C). Quercetin-stimulated increases in CBF were also inhibited by blocking NO signaling with L-NAME (10 µM; Figure 6D). These data suggest that quercetin activation of CBF may occur through T2R activation and NO production. 

**Figure 7:**
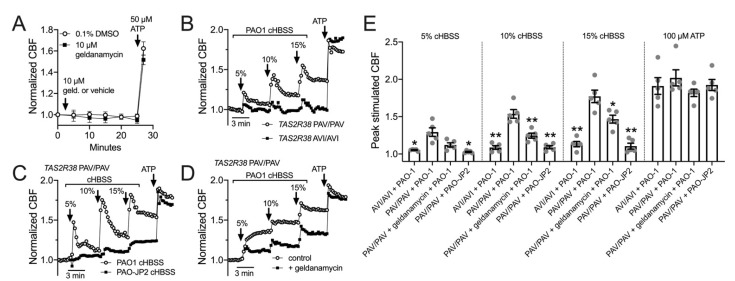
HSP90 inhibition reduces epithelial ciliary response to *P. aeruginosa* conditioned medium. (**A**): Graph shows real-time measurement of CBF (mean ± SEM of six independent experiments using ALIs from three patients) during prolonged geldanamycin treatment, followed by stimulation with purinergic agonist ATP. (**B**): Primary nasal ALIs genotyped for functional T2R38 (TAS2R38 PAV/PAV) or non-functional T2R38 (TAS2R38 AVI/AVI) were stimulated with diluted HBSS in which *P. aeruginosa* PAO-1 had been incubated overnight (conditioned HBSS; cHBSS, diluted with unconditioned HBSS). Peak CBF responses to PAO-1 cHBSS were greater in PAV/PAV cells vs. AVI/AVI cells. Representative trace shown from five experiments using cultures from separate individual patients. (**C**): PAV/PAV cells were stimulated with cHBSS from PAO-1 or PAO-JP2, which lacks the ability to produce AHLs. PAO-1 cHBSS stimulated CBF increases that were greater than CBF increases observed with PAO-JP2 cHBSS. Representative trace shown from five experiments using cultures from separate individual patients. (**D**): PAV/PAV cells were stimulated with PAO-1 cHBSS ± geldanamycin pretreatment. Representative trace shown from five experiments using cultures from separate individual patients. (**E**): Bar graph showing peak CBF (mean ± SEM with individual data points showing individual experiments) observed from experiments as in *F-H*. Asterisks represent significance compared with PAV/PAV + PAO-1 cHBSS at each individual concentration, determined by Sidak’s multiple comparison test; * *p* < 0.05 and ** *p* < 0.01.

Importantly, we observed that geldanamycin has no significant effect on baseline CBF after ≥20 min (Figure 7A). This is in contrast to prior studies in mouse tracheal cells, where geldanamycin rapidly reduced CBF to ~75% of basal values, postulated to be due to reduced stability of tubulin polymerization upon HSP90 inhibition [55]. We did not see these effects here in human nasal cultures.

We also tested CBF response to HBSS that had been conditioned by overnight exposure to *P. aeruginosa*. We previously performed similar experiments with conditioned LB medium and showed that CBF increases in response to dilute (6.25–12%) *P. aeruginosa* medium was dependent on bitter receptor T2R38, which is expressed in cilia and detects acylhomoserine lactone (AHL) quorum sensing molecules [34]. Here, *P. aeruginosa* Wt strain PAO-1 was incubated in HBSS for 24 h, and the resulting conditioned HBSS (cHBSS) was diluted and used to stimulated cells. We found that 5–15% cHBSS stimulated robust ciliary responses in nasal ALIs homozygous for the functional polymorphism (PAV) of the TAS2R38 gene encoding the T2R38 receptor (Figure 7B). Cells homozygous for the non-functional (AVI) polymorphism of TAS2R38 responded with much lower CBF increases (Figure 7B), showing the responses were dependent on T2R38. With cHBSS from strain PAO-JP2, which is unable to produce AHLs [65,66,100], we observed minimal CBF responses in PAV/PAV cells compared with PAO-1 Wt cHBSS (Figure 7C), showing the response were dependent on AHL signaling. Notably, AHL signaling also control production of quinolone quorum sensing molecules [101], which can also function as T2R agonists [5,22]. Fitting with a role for HSP90 in T2R function, we observed that geldanamycin reduced the CBF response to PAO-1 cHBSS in PAV/PAV cells (Figure 7D). These data are summarized in Figure 7E and together suggest that geldanamycin can reduce the ability of nasal ALIs to detect *P. aeruginosa* through T2Rs and increase ciliary beating.

### 3.6. HSP90 Inhibition Reduces T2R/NO-Driven Bacterial Killing

**Figure 8:**
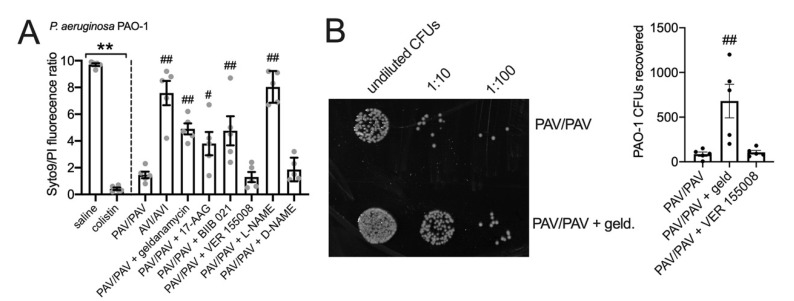
HSP90 inhibition reduces nasal epithelial bacterial killing mediated by T2Rs and NO. *P. aeruginosa* PAO-1 bacteria were incubated with nasal ALI cultures as described in the methods. (**A**): Bar graph showing live (Syto9)/dead (propidium iodide [PI]) staining quantified by fluorescence plate reader. First two bars represent bacteria incubated in the absence of nasal cells treated with saline only or saline + colistin. This illustrates max (saline) and min (colistin) live/dead ratios. Significance by one way ANOVA with Bonferroni post-test; ** *p* < 0.01 between bracketed groups; ^#^ *p* < 0.05 and ^##^ *p* < 0.01 vs. PAV/PAV cultures with no inhibitor. (**B**): Representative image (left) and bar graph (right) showing CFU counts from experiments as shown in (**A**). HSP90 inhibitor geldanamycin reduced bacterial killing (increased CFUs) while HSP70 inhibitor VER 155008 did not. Significance by one-way ANOVA with Dunnett’s post test comparing all values to PAV/PAV control (no inhibitor); ^##^ *p* < 0.01 vs. PAV/PAV control.

We used an antibacterial killing assay to test the T2R/NO-dependent bacterial killing of nasal ALI cultures [34]. Two-hour incubation of P. aeruginosa strain PAO1 with nasal ALIs results in bacterial killing that is dependent on T2R38 genotype, as PAV/PAV (functional T2R38) cultures kill bacteria while AVI/AVI (non-functional T2R38) do not (Figure 8A). This occurs because acyl-homoserine lactones (AHLs) in the medium activate T2R38, causing NO production that is bactericidal [34]. Bacterial viability was quantified by a live/dead (Syto9/propidium iodide) stain (Figure 8A) and verified by CFU counting (Figure 8B). NOS inhibitor L-NAME but not inactive D-NAME inhibited bacterial killing (Figure 8A). HSP90 inhibitors geldanamycin, 17-AAG, or BIIB 021 all reduced bacterial killing (Figure 8A,B) while HSP70 inhibitor VER155008 had no effect. These data suggest that HSP90 inhibition reduces airway innate immunity by reducing both bacterial clearance (driven by ciliary beating) and by lowering bactericidal NO production. 

### 3.7. HSP90 Inhibition Reduces T2R NO Production and Phagocytosis in Primary Human MΦs 

**Figure 9:**
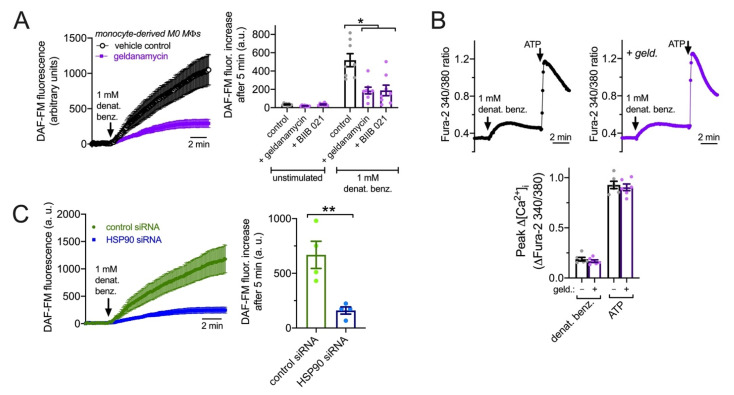
HSP90 inhibition reduces T2R-stimulated NO production in primary human M0 MΦs. (**A**): DAF-FM-loaded MΦs exhibited increases in fluorescence in response to 1 mM denatonium benzoate that were strongly inhibited by geldanamycin. Left shows average traces and right shows bar graphs (mean ± SEM) from eight independent experiments using MΦs from two donors. DAF-FM fluorescence increase was also inhibited by BIIB 021. Control = denatonium benzoate after pretreatment with 0.1% DMSO. Significance by one way ANOVA with Bonferroni posttest; * *p* < 0.05. (**B**): Low-level Ca^2+^ responses to denatonium benzoate were not affected by geldanamycin. Top shows representative traces in the absence or presence of 1 µM geldanamycin. Bottom shows bar graph of six independent experiments using MΦs from three different donors. Response to purinergic agonist ATP shown as control. (**C**): NO production in MΦs treated with HSP90 or control non-targeting siRNAs. Left shows representative traces and right shows bar graph of data from four independent experiments per condition. Significance by Student’s *t*-test; ** *p* < 0.01.

We wanted to examine T2R signaling to eNOS in another human primary cell model to test if it requires HSP90 function. Like epithelial cells, MΦs are important players in early innate immunity. Unprimed (M0) monocyte-derived MΦs also express eNOS involved in enhancement of phagocytosis during immune receptor activation [23]. While isolated monocytes differentiate into MΦs that are not exactly the same as alveolar MΦs that populate the airways at baseline [102,103,104], monocyte-derived MΦs are often used as surrogates for alveolar MΦs and are nonetheless themselves important for infections, including during chronic airway inflammation such as CRS, chronic obstructive pulmonary disease (COPD), asthma, and cystic fibrosis [15,105,106]. We previously observed that T2R stimulation in human M0 monocyte-derived MΦs also activates low-level Ca^2+^ responses that drive NO production to enhance phagocytosis [31]. Macrophage DAF-FM responses to denatonium benzoate were significantly reduced by HSP90 inhibitors geldanamycin and BIIB 021 (10 µM; 30 min pretreatment; Figure 9A) despite no change in denatonium-induced Ca^2+^ signals (Figure 9B), suggesting that HSP90 is required for activation of eNOS and/or nNOS downstream of the T2R-induced Ca^2+^ response [31]. To confirm this, we treated MΦs with pooled siRNAs directed against HSP90 or with non-targeting scrambled control siRNAs. We found that HSP90 siRNAs reduced the NO production driven by T2R agonist denatonium benzoate compared with control siRNAs (Figure 9C). Both pharmacological inhibition and genetic knockdown thus suggest that HSP90 is required for maximal NO production downstream of T2Rs in MΦs. 

**Figure 10:**
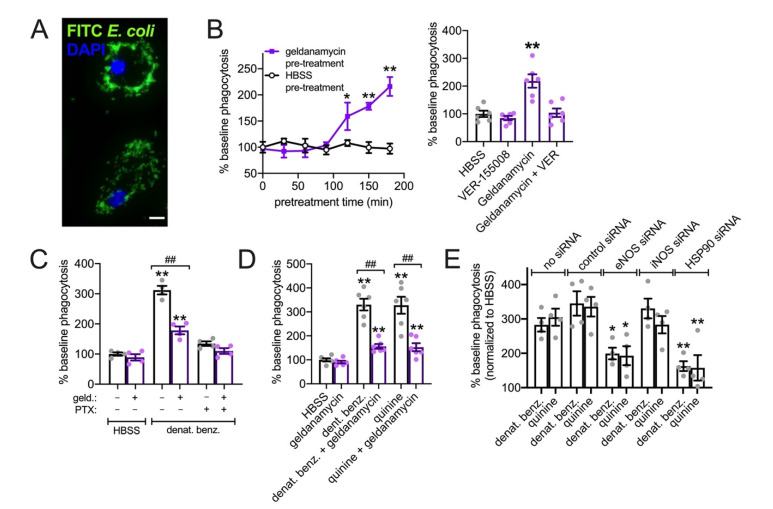
HSP90 inhibition reduces T2R-stimulated FITC-*E. coli* phagocytosis in primary human M0 MΦs. (**A**): Representative image of MΦs with phagocytosed FITC-labeled *E. coli*. (**B**): Left shows time course of phagocytosis responses during 30 min incubation in HBSS as described in the methods after pretreatment with geldanamycin or HBSS for times indicated on the y-axis. Each data point is the mean ± SEM of three independent experiments using MΦs from three different donors. Right shows separate experiments of baseline phagocytosis over 30 min (HBSS only) of FITC-*E. coli* after 2 h pretreatment with HBSS only (containing 0.1% DMSO as vehicle control), 1 µM VER-15508, 1 µM geldanamycin, or geldanamycin plus VER-15508. Significance determined by one-way ANOVA with Dunnett’s post-test comparing values to HBSS pretreatment; * *p* < 0.05, ** *p* < 0.01. Bar graph shows the mean ± SEM of six experiments using MΦs from three donors. (**C**): Stimulated 30 min phagocytosis of FITC-*E. coli* (HBSS only control or 1 mM denat. benz. ± pertussis toxin [PTX]) was measured after pre-incubation with HBSS + 0.1% DMSO or 1 µM geldanamycin. PTX and geldanamycin both inhibited denatonium-induced phagocytosis. Significance determined by one-way ANOVA with Bonferroni post-test; ** *p* < 0.01 vs. HBSS control and ^##^ *p* < 0.01 vs. bracketed groups. (**D**): Geldanamycin reduced phagocytosis increases observed with both denatonium and quinine. Bar graph shows the mean ± SEM of six independent experiments using cells from six different individual patients. Significance by one way ANOVA with Tukey–Kramer post-test comparing all bars; ** *p* < 0.01 vs. HBSS alone; ^##^ *p* < 0.01 vs. bracketed bar. (**E**): Assays were carried out in MΦs previously treated with siRNAs directed against eNOS, iNOS, HSP90, or non-targeting control sequences. Bar graph shows increase in phagocytosis relative to HBSS in the same macrophage background over four independent experiments. Significance compared with no siRNA control using one-way ANOVA with Bonferroni post-test and pairwise comparisons; * *p* < 0.05 and ** *p* < 0.01.

We measured phagocytosis of FITC-labeled *Escherichia coli* (Figure 10A). First, we had to test effects of geldanamycin alone. It was previously shown that geldanamycin treatment and HSP90 inhibition increase phagocytosis after ~90 min due to transcriptional up-regulation of HSP70 [107,108]. We observed an increase in baseline phagocytosis after ~2 h geldanamycin treatment (Figure 10B); this was inhibited by HSP70 inhibitor VER-155008 (Figure 10B), supporting these prior observations. Thus, to avoid any effects of HSP70 up-regulation, we tested the effects of geldanamycin on denatonium-upregulated phagocytosis after only 30 min geldanamycin pretreatment followed by continued geldanamycin treatment for the 15 min of the phagocytosis assay (45 min total). We observed a ~3-fold increase in phagocytosis in response to 1 mM denatonium benzoate (as we previously reported [31]) that was inhibited by geldanamycin as well as pertussis toxin (Figure 10C), which inactivates the G_i_ and G_gustducin_ Gα subunits that can couple to T2R receptors [98,109,110]. We also saw inhibition of denatonium benzoate-induced or quinine (500 µM)-induced phagocytosis of pHrodo-labeled *S. aureus* with geldanamycin pretreatment (Figure 10D). Together, these data suggest that HSP90 plays a key role in activation of NO production downstream of T2R activation. We found that knockdown of either eNOS or HSP90 by siRNA also reduced phagocytosis activated by T2R agonists denatonium benzoate or quinine (Figure 10E). As a control, knockdown of iNOS had not effect (Figure 10E). Thus, the importance of HSP90 in this process is supported by both pharmacological and genetic approaches. 

To confirm that our FITC-*E. coli* measurements reflected phagocytosis and to test a pathogen with more relevance to the airway epithelium, we also tested pHrodo-labeled *Staphylococcus aureus*, as previously utilized in [31]. The pHrodo dye fluorescence reacts strongly in acidic environments and thus exhibits a marked increase in fluorescence when internalized into acidic organelles such lysosomes and phagosomes. Assays were carried out similarly to FITC-*E. coli* assays described above.

**Figure 11:**
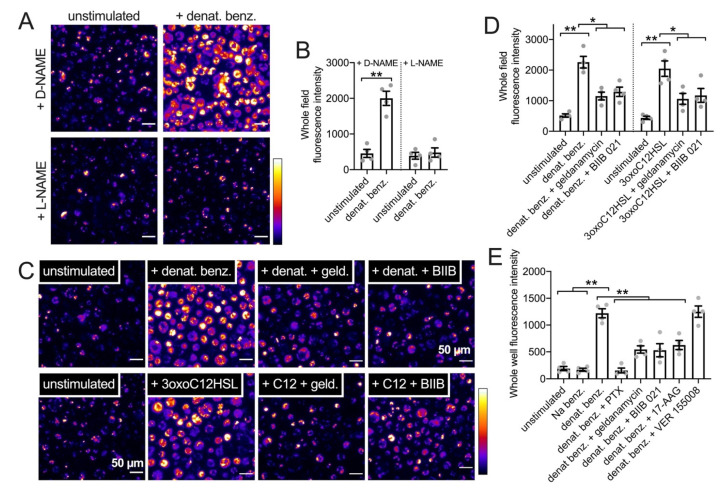
HSP90 inhibition reduces T2R-stimulated pHrodo-*S. aureus* phagocytic responses in primary human M0 MΦs. (**A**): Representative images of pHrodo-labeled *S. aureus* phagocytosis in primary human MΦs ± denatonium benzoate (1 mM) stimulation after D-NAME or L-NAME pretreatment (10 µM; 45 min). (**B**)*:* Bar graph of pHrodo-*S. aureus* fluorescence after experiments as in *A*. Significance by Bonferroni post-test with paired comparisons; ** *p* < 0.01. (**C**): Representative images of pHrodo-labeled *S. aureus* phagocytosis in primary human MΦs ± denatonium benzoate (1 mM) or 3oxoC12HSL (100 µM) after no-pretreatment (0.1% DMSO only as vehicle control)) or pretreatment with HSP90 inhibitors geldanamycin or BIIB 021 (pretreatment as in Figure 8). (**D**): Bar graph of pHrodo-*S. aureus* phagocytosis during stimulation with HBSS only (unstimulated control), 1 mM denatonium benzoate, or 100 µM 3oxoC12HSL ± geldanamycin or BIIB 021 (pretreatment as in Figure 9). Significance by one-way ANOVA with Bonferroni post-test; * *p* < 0.05 or ** *p* < 0.01. (**E**): Bar graph of pHrodo-*S. aureus* phagocytosis during stimulation with HBSS only (unstimulated control) or 1 mM denatonium benzoate ± pertussis toxin (PTX), geldanamycin, BIIB 021, 17-AAG, or VER 15508. PTX (500 ng/mL) pretreatment was 18 h. MΦs were pretreated with other inhibitors as in Figure 9. Significance by one-way ANOVA with Bonferroni post-test; ** *p* < 0.01.

We observed that denatonium benzoate (1 mM) increased phagocytosis in a NOS-dependent manner as it was inhibited by L-NAME but not D-NAME (10 µM, 30 min pretreatment; Figure 11A,B). The increased phagocytosis in response to denatonium benzoate or *P. aeruginosa* 3oxoC12HSL (100 µM) was inhibited by geldanamycin or BIIB 021 (10 µM pretreatment; Figure 11C,D), supporting a reduction in this innate immune response by HSP90 inhibitors. We tested other HSP90 inhibitors using pHrodo *S. aureus* in the same assay in a plate reader format (as described in the methods and [31]). Well fluorescence increased when MΦs were incubated with 1 mM denatonium benzoate for 15 min (Figure 11E). Equimolar sodium benzoate had no effect (Figure 11E). The stimulatory effect of denatonium benzoate was reduced by pertussis toxin (to block T2R GPCR signaling) or pre-incubation (15 min; 10 µM) with HSP90 inhibitors geldanamycin, BIIB 021, or 17-AAG but not HSP70 inhibitor VER 155008 (Figure 11E).

## 4. Discussion

HSP90 likely plays a multi-faceted role in airway epithelial physiology beyond facilitating protein folding, but data surrounding its specific contributions are unclear. There has been an increasing interest in heat shock chaperone proteins in immune cell modulation [111], including regulation of immune cell metabolism [112] and immune receptor signaling [113,114]. Here, we show a new innate immune role for HSP90, namely the production of NO downstream of T2R signaling. Specifically, we show that HSP90 inhibition by multiple structurally diverse compounds acutely impairs NO-mediated airway epithelial CBF responses and macrophage phagocytosis without impairing the upstream calcium signaling. Thus, the result of HSP90 inhibition is not simply impaired receptor function, trafficking, or folding. We utilized several models, from heterologous expression in HEK293T and A549 cells to human primary cells differentiated from patient material. 

The T2R to eNOS pathway, specifically polymorphisms regulating T2R38 signaling, has been identified as clinically important in terms of increased susceptibility to upper respiratory infections and impaired patient outcomes in chronic rhinosinusitis (CRS) [35,36,37,38,40,41,42,115,116,117]. T2R signaling to eNOS regulates both airway ciliary beating and macrophage phagocytosis. Others have shown that HSP90 is important for scaffolding eNOS with activating proteins such as Akt or Ca^2+^-bound calmodulin. We hypothesize that this scaffolding function is likewise important for T2R activation of eNOS. This puts HSP90 in a prime role to regulate eNOS output during T2R stimulation. HSP90 transcript levels can be regulated by a host of transcription factors active during inflammation or cell stress, including HSF1, NF-IL6, and NFκB [118]. Various post-transcriptional modifications, from phosphorylation to nitrosylation [48,118,119], can also alter HSP90 activity. We hypothesize that regulation of HSP90 may be one way to modulate airway T2R/eNOS NO output or airway NO output in general. In a myocardial ischemia–reperfusion injury mouse model, transfection of HSP90 is protective by enhancing eNOS S1177 (activating site) phosphorylation and decreasing eNOS T495 (inhibitory site) phosphorylation [120]. We hypothesize that HSP90 expression might be a pathway that could be exploited to increase NO in airway diseases associated with reduced NO levels, including CF [121,122,123,124,125,126,127,128,129] or primary ciliary dyskinesia [130,131,132].

While HSP90 has been shown to be important for baseline motile cilia function [48,54,55] as well as NO-driven alcohol-stimulated cilia function [48], other studies have suggested that HSP90 is necessary for Th2 (IL-13-driven) and Th17 (IL-17-driven) airway goblet cell metaplasia [56]. Many airway diseases, including asthma, COPD, and CF, are characterized by a loss of ciliated cells due to goblet or squamous metaplasia, likely impairing mucociliary clearance both through mucin hypersecretion and loss of cilia [133]. HSP90 inhibition was suggested to be potentially useful in type 2 inflammatory airway disease characterized by airway remodeling, typically goblet cell metaplasia [56], which include asthma and chronic rhinosinusitis. However, outside the airway, HSP90 has been implicated in both pro-inflammatory and anti-inflammatory processes [111]. A better understanding of how HSP90 contributes to the myriad of functions that airway epithelial cells perform, including bacterial surveillance and antimicrobial responses, is needed.

While HSP90 is required for NO production during stimulation of airway and macrophage T2Rs, the inhibition of HSP90 did not affect T2R-mediated Ca^2+^ signals upstream of the NO production. HSP90 inhibition reduced T2R-mediated cGMP production and reduced T2R-mediated elevation of ciliary beating, bacterial killing, and phagocytosis, all downstream of NO production. Thus, HSP90 inhibition may reduce innate immune responses to bacteria in the airway, through both epithelial cells and dedicated immune cells. The overall effect on airway innate immunity will depend on other pathways that are up or down regulated. It may be that the reversal of goblet metaplasia in asthma with HSP90 inhibition outweighs a side-effect of reduced T2R responses, but the knowledge that these T2R responses are reduced may suggest other supplemental targets/therapies are needed to boost NO production in patients receiving HSP90 inhibitors. Here, we simply show one effect of HSP90 inhibition that would be predicted to be detrimental. Other effects of HSP90 inhibitors in the airway must be studied in more detail to clarify the entire picture of how these drugs may affect the respiratory epithelium and innate defense. 

As described above, HSP90 has been localized to the base of airway cilia [48,54] in close proximity to eNOS [18,21,48,134] and T2Rs [4,34]. We found that T2R activation produces more NO in ciliated airway cells than stimulation of purinergic receptors [34] or PAR-2 [135], despite the fact that these other GPCRs generate higher Ca^2+^ responses than T2Rs. A clearer picture of the T2R signaling pathway is necessary to understand why this occurs. It may be that the close proximity of T2Rs to eNOS and HSP90 creates localized Ca^2+^ or calmodulin microdomains within the cilia. Another explanation is that other T2R-stimulated pathways downstream or in parallel to the Ca^2+^ also contribute to these responses. While eNOS can be activated directly through interactions with Ca^2+^-bound calmodulin [136,137], it can also be activated by phosphorylation at multiple residues by kinases such as Akt, CaMKII, or PKA [138,139,140,141,142,143,144,145,146,147]. Phosphorylation at one or more eNOS residues may be important during T2R activation of NO production. While much work on T2Rs has focused on Ca^2+^ activation downstream of the Gβγ component of their heterotrimeric G protein signal pathway, little is known about kinases activated during T2R stimulation. Future studies are needed to better elucidate the molecular mechanisms of T2R signaling to eNOS in airway epithelial cells and other cells such as MΦs.

## Data Availability

Data are contained within this article or Supplementary Materials. Raw numerical values used to generate bar graphs or traces are available upon request.

## Supplementary Materials

The following supporting information can be downloaded at: https://www.mdpi.com/article/10.3390/cells11091478/s1, Figure S1: Role of HSP90 in NO production by heterologously expressed endothelial nitric oxide synthase (eNOS) in HEK293T cells; Figure S2: Role of HSP90 in NO production by heterologously expressed endothelial nitric oxide synthase (eNOS) in A549 cells; Figure S3: eNOS, T2R4, and T2R46 immunofluorescence in submerged subconfluent H441 cells; Figure S4: T2R agonist denatonium benzoate activates HSP90-dependent NO production in submerged H441; Figure S5: Geldanamycin inhibits NOS-dependent cGMP responses during denatonium benzoate stimulation in H441 cells.
