# Supplementary material for: HSP90 Modulates T2R Bitter Taste Receptor Nitric Oxide Production and Innate Immune Responses in Human Airway Epithelial Cells and Macrophages
Source: Cells. 2022 Apr 27;11(9):1478. doi: 10.3390/cells11091478 (PMC9101439; doi:10.3390/cells11091478)
Supplement: Supplementary file 1 [file cells-11-01478-s001.zip › cells-1686253-supplementary.pdf]

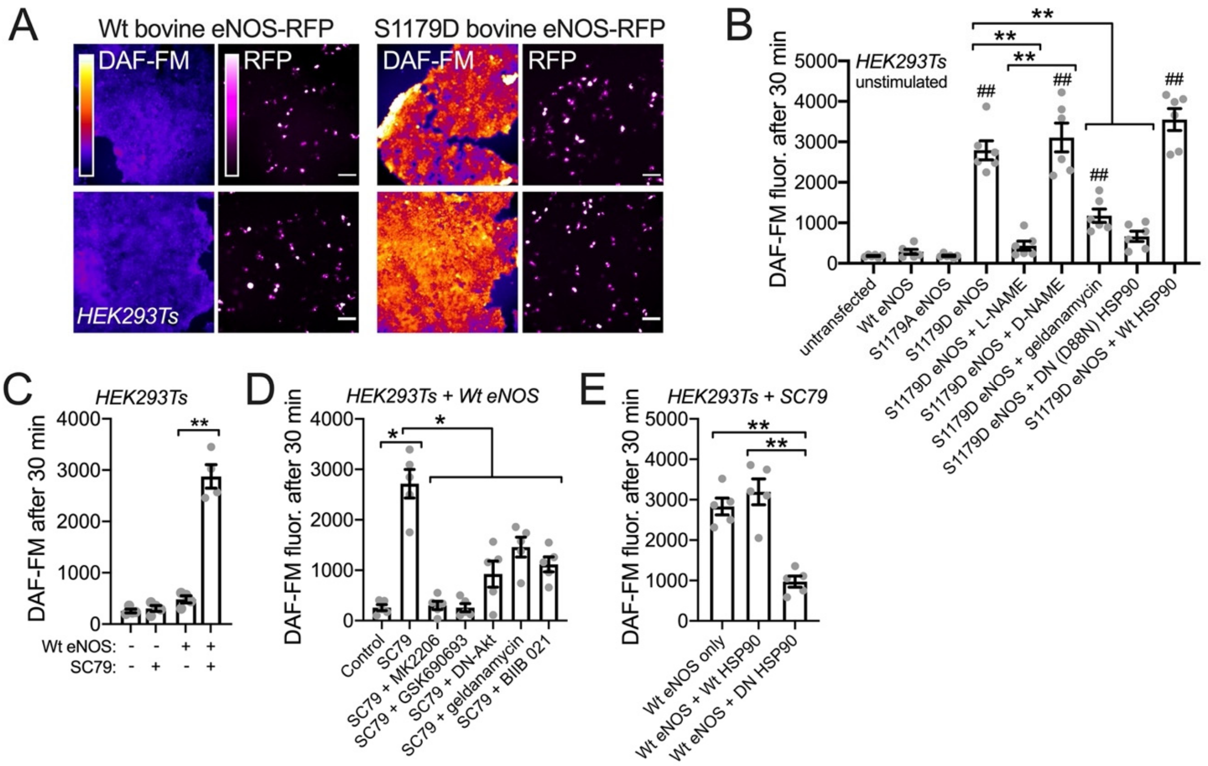

Figure S1. Role of HSP90 in NO production by heterologously-expressed endothelial nitric oxide synthase (eNOS) in HEK293T cells. ***A:*** Live-cell images of HEK293T cells expressing bovine eNOS-RFP (either wild type [Wt] or S1179D) after 30 min loading with DAF-FM by incubation in cell permeant DAF-FM diacetate. ***B:*** Bar graph of cell fluorescence intensity from experiments as in *B*. S1179D eNOS expression increased DAF-FM fluorescence over Wt or S1179A eNOS. DAF-FM fluorescence increase with S1179D eNOS was inhibited by NOS inhibitor L-NAME, HSP90 inhibitor geldanamycin, or dominant negative (DN) D88N HSP90. Bar graph is mean ± SEM of n = 6 independent experiments, shown by data points. Significance by Bonferroni posttest; ***p*<0.01 vs bracketed groups and ##*p*<0.01 vs transfected control. Note that DAF-FM fluorescence increases likely reflected NO production as they were inhibited after 30 min pre-treatment and in the continued presence of 10 μM L-NAME but not equimolar inactive control D-NAME. **C:** In HEK293Ts expressing Wt eNOS-RFP, SC79 increased DAF-FM fluorescence after 30 min loading. Bar graph shows mean ± SEM of 4 independent experiments. Significance by Bonferroni posttest comparing untransfected ± SC79 and comparing eNOS transfected ± SC79; ** *p*<0.01. ***D:*** In HEK293Ts expressing Wt eNOS, SC79 increased DAF-FM fluorescence after 30 min loading, which was reduced with Akt inhibitors MK2206, GSK690693 (10 μM; 30 min pre-treatment then continued throughout the 30 min experiment), co-transfection of dominant negative (DN)-Akt (K179M Akt), or HSP90 inhibitors geldanamycin or BIIB 021. Bar graph shows mean ± SEM (n = 5 independent experiments). Significance by Bonferroni posttest with paired comparisons; **p*<0.05. ***E:*** In HEK293Ts stimulated with SC79 (10 μg/ml) and transfected with Wt eNOS, co-transfection of DN HSP90 but not Wt HSP90 reduced DAF-FM fluorescence after 30 min loading. Bar graph shows mean ± SEM (n = 5 independent experiments). Significance by one way ANOVA with Bonferroni posttest; ***p*<0.01.

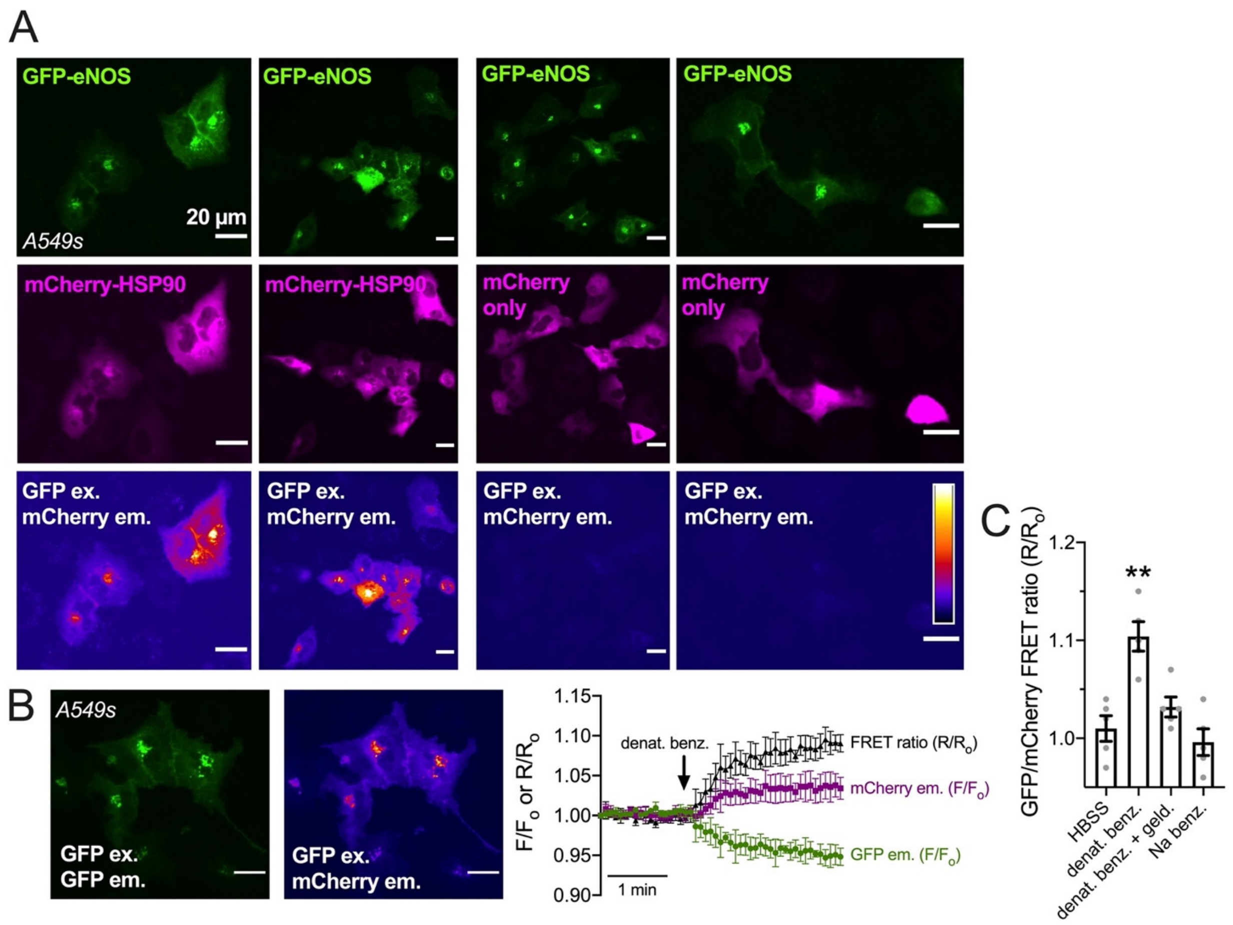

Figure S2. Role of HSP90 in NO production by heterologously-expressed endothelial nitric oxide synthase (eNOS) in A549 cells ***A:*** A549 cells were chosen here because of their transfectability and their ability to stick well to glass and spread out for imaging. Note that A549 cells are not ciliated. Representative images (3 independent transfection experiments) of A549 cells transfected with GFP-eNOS (green) and mCherry HSP90 (magenta; left 2 columns) or mCherry alone (magenta; right 2 columns). Intensity pseudocolored images at the bottom show fluorescence with GFP excitation filters and mCherry emission filter, shown at identical microscope settings for all 4 columns. We saw punctate perinuclear localization of eNOS likely reflecting Golgi, as eNOS localizes partly to the Golgi in endothelial cells [1] (top row, first two columns). We also saw some likely plasma membrane eNOS localization at cell-cell contact points (note first and second columns), also expected from studies of endothelial cells [2]. HSP90 localization, in contrast, was more global (middle column), though some perinuclear intensity was observed. However, when we excited GFP (eNOS) and collected mCherry (HSP90) emission, we saw an mCherry emission signal that appeared identical to the GFP-eNOS localization (compare top row to bottom row). As GFP and mCherry are a donor-acceptor pair for Förster resonance energy transfer (FRET), we hypothesized that we were collecting FRET emission from mCherry-HSP90 in close proximity to the excited GFP-eNOS. When this experiment was performed with GFP-eNOS and mCherry alone (no linked HSP90), no mCherry emission was detected with GFP excitation (last two columns). ***B:*** A549 cells endogenously express T2R bitter taste receptors activated by the bitter agonist denatonium benzoate [3]. When we monitored GFP and mCherry emission, both with GFP excitation, we noted an increase in mCherry emission and concomitant decrease in GFP emission with denatonium benzoate stimulation, suggesting that an increase in FRET occurs with bitter agonist stimulation. Representative image showing 3 A549 cells transfected with GFP-eNOS and mCherry-HSP90 imaged with GFP filters (left) or GFP excitation and mCherry emission filters (right). Trace on the right shows normalized (F/F_o) average of GFP vs mCherry emission (both using GFP excitation) as well as the ratio of mCherry/GFP emission (FRET ratio; R/R_o). Note that 1 mM denatonium benzoate increases mCherry (acceptor) emission but decreases GFP (donor) emission, suggesting a bona fide increase in FRET. ***C:*** This increase in FRET was not observed during stimulation with sodium benzoate (Na benzoate) and was reduced with HSP90 inhibitor geldanamycin. Bar graph showing mean ± SEM of R/R_o from independent experiments (n = 5) as in G. Cells were stimulated with HBSS only (control),

sodium benzoate (1 mM), or denatonium benzoate (1 mM) ± geldanamycin (10 μM; 30 min pre-treatment plus stimulation with continued 10 μM geldanamycin). HBSS, denat. benz., and Na benzoate conditions contained 0.1% DMSO as vehicle control. Significance by one way ANOVA with Dunnett's posttest comparing all values to HBSS alone; ***p*<0.01.

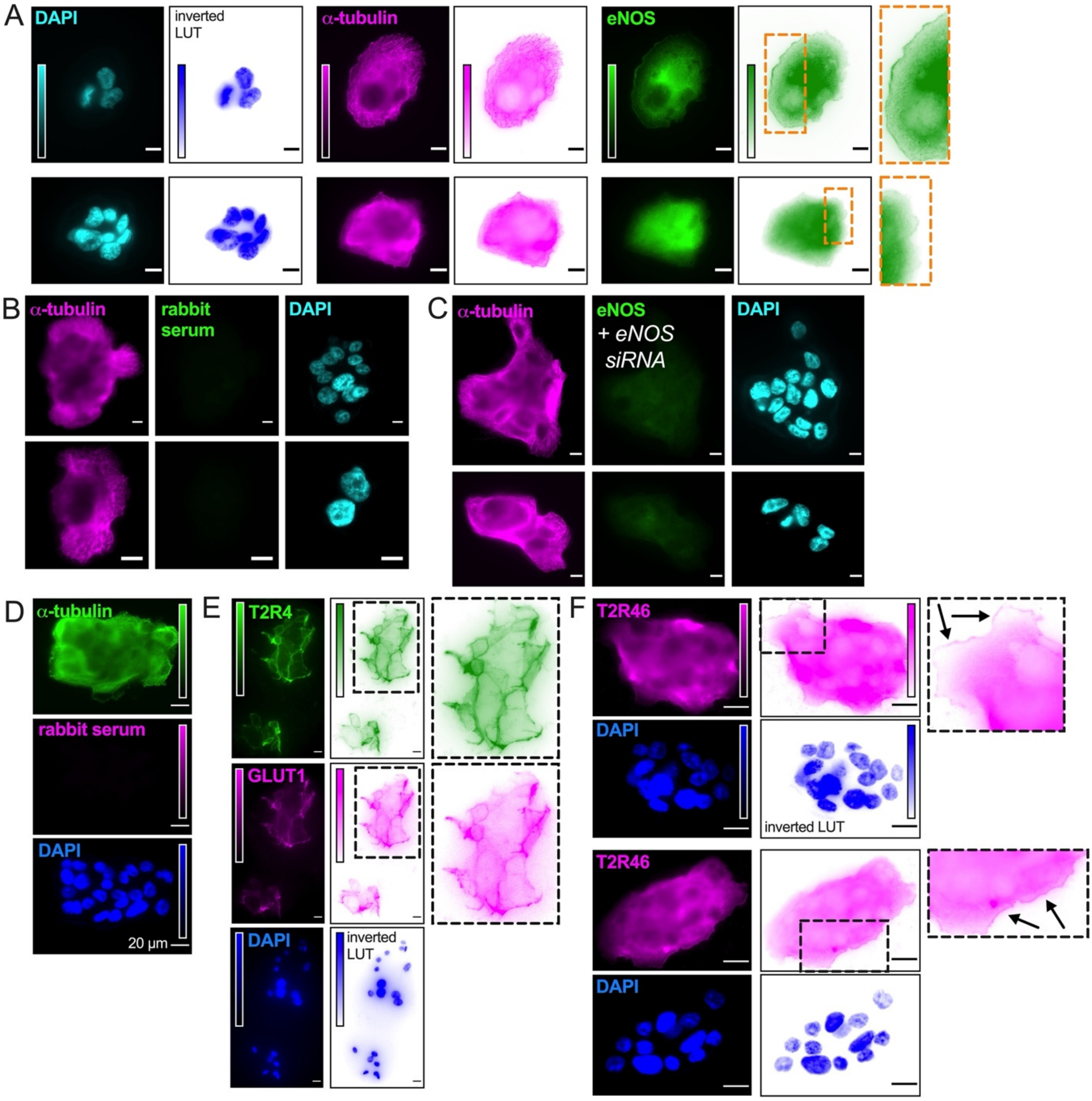

Figure S3. eNOS, T2R4, and T2R46 immunofluorescence in submerged sub-confluent H441 cells. ***A*:** Immunofluorescence images of cells stained with primary mouse monoclonal antibody for α-tubulin and rabbit polyclonal antibody to eNOS. Inverted look-up table (LUT) images are shown along with conventional LUTs to highlight localization. ***B*:** Rabbit serum plus secondary antibody control. Cells were stained, fixed, and incubated in parallel with cells from experiments as in (A), and imaged at identical microscope settings (objective, exposure time, *etc.*). ***C*:** This eNOS signal was blocked by pre-treatment of H441 cells with eNOS siRNA. Images of eNOS siRNA-treated cells stained with primary mouse monoclonal antibody for α-tubulin and rabbit polyclonal antibody to eNOS, imaged at identical microscope settings to A and B. ***D-E*:** Images of rabbit serum only (D) or T2R4 (E) localization, showing similar pattern of T2R4 and GLUT1 staining (D). ***F*:** T2R46

stained cells with inset regions blown up to show membrane-like staining at the edges of the epithelial cell patches. All images representative of ≥3 independent experiments. Scale bars in A-C are 10 μm. Scale bars in D-F are 20 μM. Note that T2R4 staining was similar to the pattern observed for plasma membrane glucose transporter Glut1 (E), while T2R46 appeared to be more diffusely localized but also possibly somewhat localized to the edges of some H441 cell islands (F). Like many GPCRs, a substantial amount of T2R46 immunofluorescence was located intracellularly, possibly representing ER and/or trafficking compartments. The implications for these different staining patterns are unclear, but our goal here was to test for T2R expression rather than perform detailed localization analysis. This immunofluorescence appears to support that both T2R4 and T2R46 are expressed in H441s in addition to downstream eNOS.

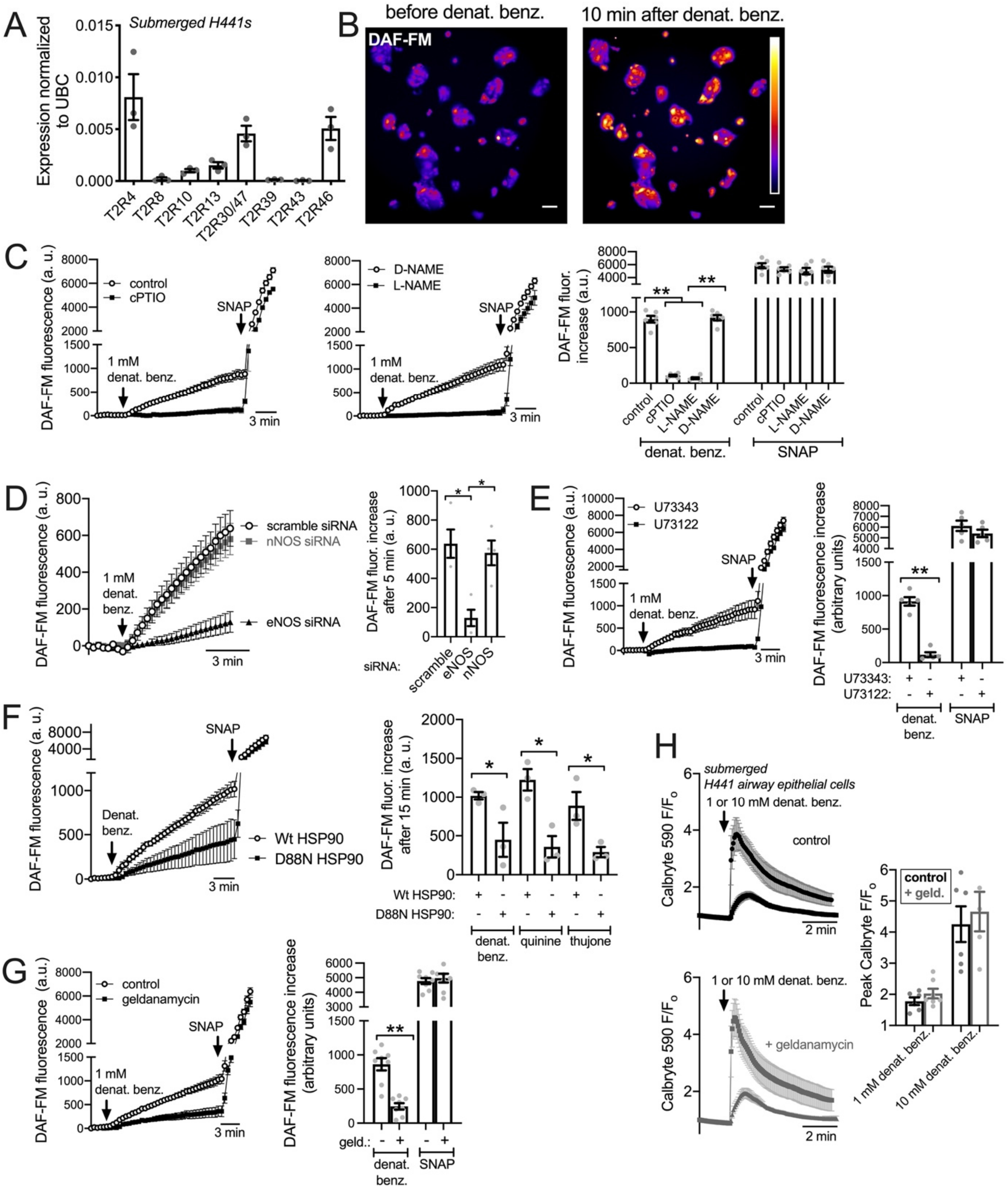

Figure S4. T2R agonist denatonium benzoate activates HSP90-dependent NO production in submerged H441. ***A:*** qPCR of denatonium-responsive *TAS2R* gene expression in H441 cells compared to UBC expression. Bar graph shows mean ± SEM of n = 3 experiments. ***B:*** Representative images of DAF-FM-loaded subconfluent H441 cells immediately before and 10 min after stimulation with 1 mM denatonium benzoate. Each distinct cell island was treated as one region of interest within one experiment. Scale bar is 20 μm. ***C*:** Left and middle show representative traces from H441 cells stimulated with 1 mM denatonium benzoate (denat. benz.) in the presence or absence of NO scavenger carboxy-PTIO (cPTIO; 10 μM added at the beginning of the experiment), NOS-inhibitor L-NAME (45 min pre-treatment; 10 μM), or inactive D-NAME (45 min pre-treatment; 10 μM). Right is bar graph of mean ± SEM of DAF-FM fluorescence increase after 10 min from 5-8 independent experiments. Significance by Bonferroni posttest with paired comparisons; $^{**}p<0.01$. The S-nitrosothiol NO donor SNAP (20 μM) was used in these experiments as a positive control. ***D*:** Experiments as in C but done in cells treated with eNOS (NOS3) or nNOS (NOS1) siRNA. Bar graph is mean ± SEM of DAF-FM fluorescence increase after 5 min from 5-8 independent experiments. Significance by Bonferroni posttest; $^{*}p<0.05$. ***E*:** Representative DAF-FM experiments (left; each trace from single experiment showing mean ± SEM of 6-8 H441 cells regions stimulated with 1 mM denatonium benzoate [denat. benz.]) and bar graph (right; mean ± SEM) from experiments in the presence of PLC inhibitor U73122 or inactive control U73343. DAF-FM increases were inhibited by U73122 but not U73343, as observed in primary nasal cells [4] and macrophages [5]. This suggests the DAF-FM responses require PLC IP_3 generation and Ca^{2+} signaling, likely downstream of T2R GPCR activation. Bar graph shows results of DAF-FM fluorescence increases after 10 min from 5-8 independent experiments per condition. Significance by Bonferroni posttest with paired comparisons; $^{**}p<0.01$. ***F*:** Representative DAF-FM traces (left and bar graph) showing stimulation of H441 cells after transfection with Wt or dominant negative (D88N) HSP90. Significance in bar graph by Bonferroni posttest with paired comparisons; $^{*}p<0.05$. Quinine was used at 500 μM and thujone at 1 mM. Quinine activates 11 T2Rs including T2R4 and 46, while Thujone activates T2Rs 14 and 10 [6,7]. ***G*:** Representative experiment (left) and bar graph (right) from experiments as in *B-E* but done ± HSP90 inhibitor geldanamycin. Significance by Bonferroni posttest with paired comparisons; $^{**}p<0.01$. ***H:*** Left and middle shows representative Ca^{2+} responses during denatonium stimulation in the absence (left) or presence (right) of geldanamycin, imaged using Calbryte 590. Bar graph shows responses from 5-7 independent experiments per condition. No significant difference determined by determined by one-way ANOVA plus Bonferroni posttest with paired comparisons.

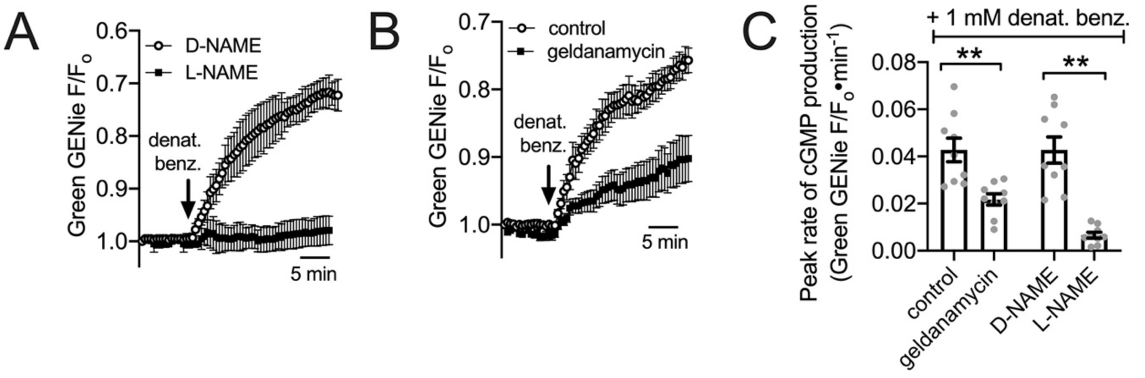

Figure S5. Geldanamycin inhibits NOS-dependent cGMP responses during denatonium benzoate stimulation in H441 cells. ***A:*** Left and middle show traces of Green GENIe cGMP biosensor experiments in H441 cells during denatonium stimulation ± D-NAME or L-NAME. Traces are plotted inversely as F/F_o goes down when cGMP goes up; thus, an upward deflection on the trace is an increase in cGMP. ***B:*** Left and middle show traces of Green GENIe cGMP biosensor experiments in H441 cells during denatonium stimulation ± geldanamycin. Control cultures were pre-treated with 0.1% DMSO as a vehicle control. ***C:*** Bar graph quantifying mean ± SEM from 6-8 independent experiments. Significance by Bonferroni posttest with paired comparisons; $^{**}p<0.01$.

Supplementary References

1. Sessa, W.C.; Garcia-Cardena, G.; Liu, J.; Keh, A.; Pollock, J.S.; Bradley, J.; Thiru, S.; Braverman, I.M.; Desai, K.M. The Golgi association of endothelial nitric oxide synthase is necessary for the efficient synthesis of nitric oxide. *J Biol Chem* **1995**, *270*, 17641-17644.
2. Sowa, G.; Liu, J.; Papapetropoulos, A.; Rex-Haffner, M.; Hughes, T.E.; Sessa, W.C. Trafficking of endothelial nitric-oxide synthase in living cells. Quantitative evidence supporting the role of palmitoylation as a kinetic trapping mechanism limiting membrane diffusion. *J Biol Chem* **1999**, *274*, 22524-22531.
3. McMahon, D.B.; Kuek, L.E.; Johnson, M.E.; Johnson, P.O.; Horn, R.L.J.; Carey, R.M.; Adappa, N.D.; Palmer, J.N.; Lee, R.J. The bitter end: T2R bitter receptor agonists elevate nuclear calcium and induce apoptosis in non-ciliated airway epithelial cells. *Cell Calcium* **2022**, *101*, 102499, doi:10.1016/j.ceca.2021.102499.
4. Carey, R.M.; Adappa, N.D.; Palmer, J.N.; Lee, R.J. Neuropeptide Y Reduces Nasal Epithelial T2R Bitter Taste Receptor-Stimulated Nitric Oxide Production. *Nutrients* **2021**, *13*, doi:10.3390/nu13103392.
5. Gopallawa, I.; Freund, J.R.; Lee, R.J. Bitter taste receptors stimulate phagocytosis in human macrophages through calcium, nitric oxide, and cyclic-GMP signaling. *Cell Mol Life Sci* **2021**, *78*, 271-286, doi:10.1007/s00018-020-03494-y.
6. Meyerhof, W.; Batram, C.; Kuhn, C.; Brockhoff, A.; Chudoba, E.; Bufe, B.; Appendino, G.; Behrens, M. The molecular receptive ranges of human TAS2R bitter taste receptors. *Chem Senses* **2010**, *35*, 157-170, doi:bjp092 [pii]10.1093/chemse/bjp092.
7. Wiener, A.; Shudler, M.; Levit, A.; Niv, M.Y. BitterDB: a database of bitter compounds. *Nucleic Acids Res* **2012**, *40*, D413-419, doi:10.1093/nar/gkr755.
